# Association of Endothelial Nitric Oxide Synthase Gene Polymorphisms with Coronary Artery Disease: An Updated Meta-Analysis and Systematic Review

**DOI:** 10.1371/journal.pone.0113363

**Published:** 2014-11-19

**Authors:** Himanshu Rai, Farah Parveen, Sudeep Kumar, Aditya Kapoor, Nakul Sinha

**Affiliations:** 1 Department of Cardiology, Sanjay Gandhi Post Graduate Institute of Medical Sciences, Lucknow, Uttar Pradesh, India; 2 Department of Medical Genetics, Sanjay Gandhi Post Graduate Institute of Medical Sciences, Lucknow, Uttar Pradesh, India; 3 Department of Cardiology, Sahara India Medical Institute, Gomti Nagar, Lucknow, Uttar Pradesh, India; St Michael's Hospital, University of Toronto, Canada

## Abstract

Several association studies of endothelial nitric oxide synthase (*NOS3*) gene polymorphisms with respect to coronary artery disease (CAD) have been published in the past two decades. However, their association with the disease, especially among different ethnic subgroups, still remains controversial. This prompted us to conduct a systematic review and an updated structured meta-analysis, which is the largest so far (89 articles, 132 separate studies, and a sample size of 69,235), examining association of three polymorphic forms of the *NOS3* gene (i.e. Glu298Asp, T786-C and 27bp VNTR b/a) with CAD. In a subgroup analysis, we tested their association separately among published studies originating predominantly from European, Middle Eastern, Asian, Asian-Indian and African ancestries. The pooled analysis confirmed the association of all the three selected SNP with CAD in three different genetic models transcending all ancestries worldwide. The Glu298Asp polymorphism showed strongest association (OR range = 1.28–1.52, and P<0.00001 for all comparisons), followed by T786-C (OR range = 1.34–1.42, and P<0.00001 for all comparisons) and 4b/a, (OR range = 1.19–1.41, and P≤0.002 for all comparisons) in our pooled analysis. Subgroup analysis revealed that Glu298Asp (OR range = 1.54–1.87, and P<0.004 for all comparisons) and 4b/a (OR range = 1.71–3.02, and P<0.00001 for all comparisons) have highest degree of association amongst the Middle Easterners. On the other hand, T786-C and its minor allele seem to carry a highest risk for CAD among subjects of Asian ancestry (OR range = 1.61–1.90, and P≤0.01 for all comparisons).

## Introduction

Endothelial-derived nitric oxide (NO) has been known to be a major contributor in vascular regulation and has been amply implicated with coronary artery disease (CAD). NO is an important relaxing factor in the human body and is involved in a variety of different important physiological functions. It is synthesized in the human body from L-arginine by at least three isoforms of NO synthase (NOS), viz. inducible NOS, constitutive neuronal NOS and constitutive endothelial NOS (eNOS). [1] NO causes vascular relaxation, it suppresses platelet and leucocyte adhesion to the vascular endothelium [2]–[4] and reduces smooth muscle cell proliferation and migration [5]. It also scavenges superoxide radicals [2] and limits the oxidation of atherogenic low density lipoproteins [3] resulting in an overall vasoprotective effect. Intracellular NO has thus been considered as an inhibitor of several key processes leading to atherosclerotic plaque formation. [4] Inhibition or reduction of NO on the other hand, may accelerate this process. NO production has been known to be influenced by several polymorphisms of the *NOS3* gene. The *NOS3* gene is located on chromosome 7q35-36, consists of 26 exons with a total size of 21 kb and it encodes for intracellular NO production [6]. This gene is expressionally and functionally regulated through multiple regulatory steps, and entails several polymorphisms [3], some of which have functional consequences.

### NOS3 polymorphisms, their role in NO production and their vascular end effects

Hingorani et al., [7] first described a point mutation of guanine (G) to thymine (T) at nucleotide 1917 in exon 7 of the *NOS3* gene, resulting in the replacement of glutamic acid by aspartic acid at codon 298 (Glu298Asp, also known as G894-T, rs1799983). This variant has been shown to be associated with coronary spasm, [8] essential hypertension [9] and the risk of acute myocardial infarction (AMI) [3], [10], [11]. Controversial results have been obtained over the years when this single nucleotide polymorphism (SNP) was evaluated with respect to its effect on intracellular NO production. Some studies have demonstrated lower concentrations of intracellular NO in genotypes carrying Asp alleles [12], while others have reported lack of association of this SNP with NO production [13]. Apparently, apart from possibly having lower NO concentrations in the human body, genotypes carrying Asp alleles have also been shown to have greater red blood cell (RBC) aggregability [14]. This makes them more prone to suffer from acute coronary events, especially from those of thrombotic origin. Befittingly, over the years this SNP has been shown to be associated to myocardial infarction (MI) [10], [11], [15], coronary artery spasm [10] and CAD. [2]–[4], [13], [16], [17] However, association of this SNP with CAD has been a matter of dispute as some studies have also reported its lack of association with the disease. [18]–[23] Geo-ethnic differences and the role of environmental factors in various populations could have been the prime reason behind these contradictory results.

Another common variant of this gene results by a cytosine replacing a thymine at nucleotide −786 (T786-C, rs2070744) at the 5′-flanking region of the *NOS3* gene. [24] The resulting T786-C variant has been shown to reduce the *NOS3* promoter activity approximately by 50% and has been associated with an increased risk for coronary artery spasm among subjects of Japanese ethnicity. [24] Recently, this polymorphism has also been shown to influence the plasma NO concentrations in hypertensive and type 2 diabetes mellitus patients. [25] It has also has been implicated as a risk factor for developing hypertension. [25] Several published reports strongly suggest that this functional variant in the *NOS3* promoter region (T786-C) is associated with reduction in the promoter efficiency and the level of expressed enzyme, leading to increased CAD risk. [26]–[28] Apart from hypertension [25], [29] and coronary artery spasm, [24] T786-C has also been investigated by several researchers for its association with the presence and severity of CAD, [3], [30] and MI. [31] However, some studies have also reported no association with CAD. [32]

There are also several variable number of tandem repeats (VNTRs) with functional significance within *NOS3* gene. Among them polymorphic repeats close to the 5′ end of the gene, the 27-base pair (bp) repeat in intron 4 is one of the most studied. The resulting rare 4-repeat allele has been shown to be associated with CAD among subjects belonging to European ancestry. [33] This 4a/b mutation of *NOS3* gene was found to be associated with MI, among subjects of Turkish descent. [34] Significant association of the 4a/b polymorphism with CAD and MI has been amply reported in several ethnic populations even after adjustment of traditional risk factors. [22], [33], [35], [36] On the other hand, a few groups have also reported lack of association of this SNP with CAD. [37]–[39]

The only meta-analysis to assess the role of all three *NOS3* SNPs with respect to CAD reported association only in recessive genetic model of Glu298Asp, while dominant and allelic comparisons came out to be non-associated in the pooled analysis (all ethnicities combined). [40] On the other hand, lack of association with CAD was seen for all three genetic models of T786-C and 4b/a. [40] Since the publication of the aforementioned study, [40] a few more recent meta-analyses focusing on either one of the selected SNPs were published reporting varied results of either association or lack of association among different employed genetic models. [41]–[44] None of them had strict criteria for inclusion of studies, which may have led to inaccurate results. Some of these published meta-analyses also included studies where genotype frequencies among controls deviated from Hardy-Weinberg equilibrium. Such deviation may hint a non-random inclusion of controls among the studies in question, which could have led to inaccurate results. Studies with several surrogate endpoints, such as “coronary artery spasm” and “heart failure”, were also included in these previously published meta-analyses. These endpoints may or may not correspond to the disease profile and pathology of CAD in general therefore such studies should have been ideally excluded. On the top of that, ethnic classifications in subgroup analyses were not appropriate in these meta-analyses and the studies were broadly and wrongly classified. We hypothesize that all these described factors may have resulted in biased effect sizes reported in all pooled as well as ethnic subgroups in these previously published meta-analyses. [40]–[44] It is also noteworthy that no genome wide association study (GWAS) published till date has identified either of these selected SNPs as risk factors for CAD. Thus, a structured meta-analysis of published association studies for *NOS3* polymorphisms, which would aid to improve the existing understanding of these polymorphic forms of this gene in relation to CAD was warranted. Taking this as an objective, we conducted a systematic review and updated meta-analysis to ascertain the role of the three most common *NOS3* gene polymorphisms in CAD. We also tested their association separately among published studies originating predominantly from European, Middle Eastern, Asian, Asian-Indian and African ancestries.

## Materials and Methods

### Literature Search

The databases of the US National Institutes of Health (PubMed), EMBASE and a MEDLINE Scopus and Web of Knowledge were systematically searched for relevant articles published online by March 2014. Both medical search headings and open text fields were used to identify articles. We systematically searched various databases and the reference lists of the relevant publications using the combination of terms like ‘NOS3’ OR ‘eNOS’ OR ‘constitutive endothelial NOS’ OR ‘ecNOS’ OR ‘endothelial nitric oxide synthase’ OR ‘endothelial NO synthase’ paired with ‘atherosclerotic heart disease’ OR ‘coronary artery disease’ OR ‘CAD’ OR ‘MI’ OR ‘Myocardial Infarction’ OR ‘AMI’ OR Acute Myocardial Infarction OR ‘ACS’ OR ‘Acute Coronary Syndrome’ AND ‘polymorphism’ OR ‘mutation’ OR ‘gene polymorphism’ Or ‘SNP’ OR ‘Single Nucleotide Polymorphism’. The search in these databases was restricted to articles relating to humans, covering all relevant English language publications published up to March 2014. All references cited in the resulting studies and in previously published reviews on this topic were examined to identify additional work not indexed in internet databases. Papers without sufficient information were identified and their corresponding authors were contacted via at least three emails (spaced one week apart) requesting needed information. Publications whose corresponding authors did not respond were thenceforth not included. Since data from unpublished studies is unlikely to be trustworthy, we chose not to include it into this present meta-analysis. The decision to include studies was hierarchical; initially study titles, then abstracts and finally the full body of the text were assessed. The following information was recorded from the retrieved studies: author's names, publication year, country where the study was conducted, study design, inclusion criteria for CAD patients and normal controls, methods used for genotyping and the distribution of polymorphic genotypes and alleles in each group.

To be included in the meta-analysis, articles had to assess the association between CAD or MI patients and CAD free controls. Our selection criteria included studies that met all of the following criteria: (1) published in a peer-reviewed journal and independent studies using original data; (2) unrelated case-control or cohort studies; (3) providing complete data with genotype and allele frequencies to calculate the odds ratio (OR) with confidence interval (CI) and p values; (4) CAD patient diagnosis based on coronary angiography/clinical assessment and controls not being CAD patients; (5) all studies included had to be published in English language; and (6) all included studies had to have their genotype distribution among controls consistent with Hardy Weinberg approximations. We excluded the case reports, case studies and the studies not providing adequate information on selection criteria and the actual distribution of genotypes in each group. We also purposefully excluded studies with a surrogate endpoint such as “coronary artery spasm” or “heart failure”. Strict adherence to the Preferred Reporting Items for Systematic Reviews and Meta-Analyses-PRISMA statement [45] and the specific recommendations for genetic meta-analysis in the HuGE Review Handbook, version 1.0 was ensured.

### Data collection and Statistical Analysis

All required data along with genotypic distribution among cases and controls were recorded from publications (in duplicate) on a paper proforma. Later allele frequency was calculated manually and all the data was then transcribed on to a Microsoft Excel program on a computer. We checked departure from Hardy-Weinberg equilibrium (HWE) among control population for each included study using Michael H. Court's (2005–2008) online calculator (http://www.tufts.edu/~mcourt01/Documents/Court%20lab%20-%20HW%20calculator.xls). Studies with genotype frequency among controls yielding a p value of <0.05, did not conform to HWE approximations and were thus excluded.

All calculations in this meta-analysis were carried out using Review Manager (*RevMan*) [Computer program]. Version 5.3. Copenhagen: The Nordic Cochrane Centre, The Cochrane Collaboration, 2012. The extracted data from all publications were tested using previously described three genetic models i.e. dominant, recessive, and allelic model. [46] Bivariate and random or fixed effect models were used for calculating effect size (i.e. Odds ratios: ORs). For each study, individual and summary ORs and its 95% confidence intervals (CIs) were calculated (for all three genetic models), using either random (DerSimonian-Laird method) [47] or fixed effects model (Mantel-Haenszel method). [48] The pooled odds ratio (OR) with a corresponding 95% confidence interval (CI) was used to assess the degree of association. Based on the individual ORs, the pooled OR was estimated, the significance of which was determined by *Z* test (P<0.05 was considered statistically significant). Summary P value and Z value was also calculated (as listed) for each study group, for each genetic model to express the significance and degree of association. Existence of heterogeneity was tested using a Q test. It was performed using Higgins *I^2^* statistics (*I^2^*) and Cochran's Q statistics (P_Q_) for each group of studies. Those resulting with *I^2^*>50% and P_Q_<0.01 were identified as a heterogeneous group. Low, moderate and high heterogeneity was defined according to previously published estimates [49] using cut off points of *I^2^* values as 25%, 50% and 75%. Using this calculation, *I^2^* value of <50% and P_Q_>0.01 was considered the criterion for selecting fixed effects for analysis. On the other hand, random effects were used for analyses for groups having *I^2^* values of >50%. In order to define the sources of heterogeneity, subgroup analysis was performed. The subgroups were classified according to predominant ancestry among which the study was conducted. Publication bias was identified by examining Begg's funnel plots [50] and by estimates from the Egger's test. [51] Sensitivity analysis was also performed in each ancestral group in every studied genetic model.

Adjustment of environmental factors is an important aspect in conducting meta-analysis of genetic association studies. However, since genetic variation among individuals is assigned at the time of birth, environmental factors like diet and lifestyle which an individual experiences thenceforth have no role in changing that attribute. [52] These factors, though can have a huge impact over the phenotype, cannot confound to the association between the genotype and the resultant phenotype. It is almost impossible to adjust environmental factors in meta-analyses of this nature. The full data is not available for every included manuscript and we have to work with only the published information. Although in an attempt to adjust the environmental factors, we stratified the studies according to predominant ethnicity among which it was conducted, which was the most we could have done.

## Results

**Figure 1:**
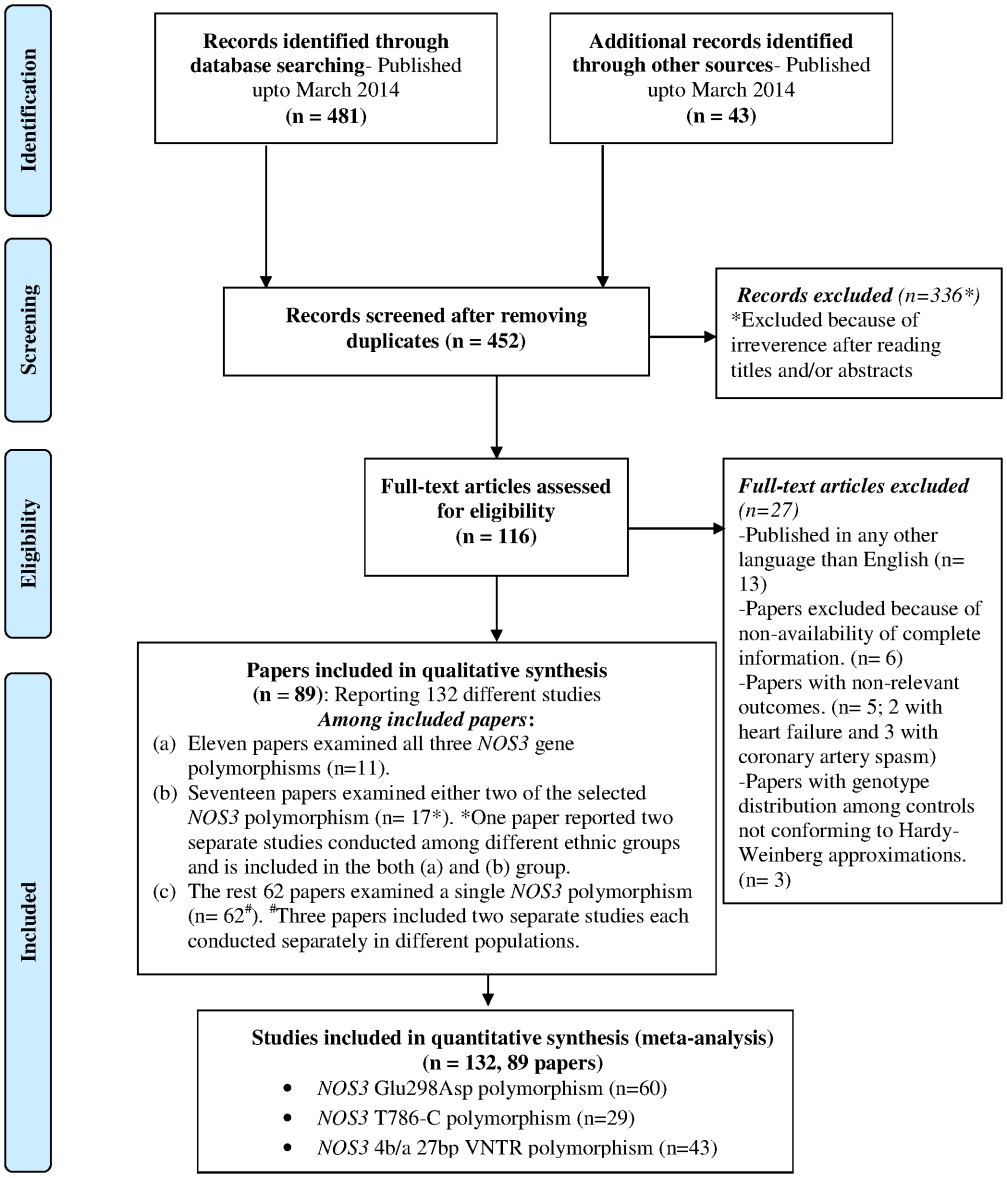
Selection of studies for inclusion.

The preliminary web search identified a total of 452 potential publications, of which 336 were immediately excluded because of obvious irrelevance after studying their titles and/or abstracts. A total of 116 articles were selected to be reviewed in full text, among which 27 articles were excluded as they met our previously described exclusion criteria. We finally selected 89 articles, which reported 132 separate studies, involving a total sample size of 69,235 to be used in the present meta-analysis. This process is explained in the form of a flowchart in **Figure 1**. The included studies were further broadly classified according to the predominant ancestry among which they were conducted. Accordingly the studies were included in one of the five ancestral groups, viz. European, Middle Eastern, Asian, Asian-Indian or African. The general characteristics of studies classified in each ancestral group, reporting association studies involving any of the three SNPs, along with an estimate of total sample tested are depicted in **Table 1**. Among these included studies, cases were generally recruited in referral centres with documented CAD or MI or ACS, and the controls were without any direct evidence of overt disease. The number of cases among all selected studies varied from 22 to 2,085, while the numbers of controls varied from 21 to 3,918.

**Table 1:** Published association studies included in the present meta-analysis.

Study	Year	Country	SNPs studied	MAF (Cases/Controls)			Total sample size (for each SNP)	Outcome
				Glu298Asp	4b/a	T786-C		
**EUROPEAN ANCESTRY GROUP**								
Wang et al. [33]	1996	Australia	4b/a	-	0.14/0.17	-	702	CAD
Cai et al. [18]	1999	Australia	Glu298Asp	0.32/0.36	-	-	763	CAD
Hingorani et al.(CHAOS) [3]	1999	UK	Glu298Asp	0.48/0.31	-	-	436	CAD
Hingorani et al.(CHAOS-2) [3]	1999	UK	Glu298Asp	0.40/0.31	-	-	432	MI
Poirier et al.(1) [38]	1999	Ireland	Glu298Asp	0.43/0.39	-	-	318	MI
Poirier et al.(2) [38]	1999	France	Glu298Asp	0.34/0.39	-	-	789	MI
Fowkes et al. [99]	2000	UK	4b/a	-	0.16/0.12	-	437	CAD
Pulkkinen et al. [100]	2000	Finland	Glu298Asp, 4b/a	0.32/0.30	0.20/0.16	-	669 for each	CAD
Sigusch et al. [101]	2000	Germany	4b/a	-	0.15/0.17	-	1043	CAD
Alvarez et al. [27]	2001	Spain	4b/a, T786-C	-	0.12/0.14	0.46/0.37	470 for each	PCAD
Granath et al. [37]	2001	Australia	Glu298Asp, 4b/a, T786-C	0.33/0.34	0.14/0.15	0.38/0.38	1194, 1187 and 1196 respectively	PCAD
Gardemann et al. [2]	2002	Germany	Glu298Asp, 4b/a	0.33/0.31	0.14/0.16	-	3250 and 3215 respectively for each	CAD
Colombo et al. [5]	2003	Italy	Glu298Asp, T786-C	0.38/0.31	-	0.51/0.40	415 and 374 respectively for each	CAD
Schmoelzer et al. [20]	2003	Austria	Glu298Asp	0.34/0.31	-	-	488	CAD
Agema et al. [54]	2004	Netherlands	Glu298Asp, 4b/a, T786-C	0.32/0.39	0.15/0.10	0.38/0.36	1329, 1218 and 1096 respectively for each	CAD
Fatini et al. [55]	2004	Italy	Glu298Asp, 4b/a, T786-C	0.39/0/33	0.19/0.15	0.48/0.39	1014 for each	ACS
Letonja et al. [102]	2004	Slovenia	4b/a	-	0.17/0.19	-	260	PCAD
Antoniades et al. [56]	2005	Greece	Glu298Asp	0.36/0.30	-	-	747	MI
Milutinovic et al. [103]	2005	Slovenia	4b/a	-	0.19/0.21	-	403	PCAD
Rao et al.(1) [104]	2005	USA	4b/a	-	0.15/0.14	-	151	CAD
Rios et al. [28]	2005	Brazil	Glu298Asp, T786-C	0.37/0.32	-	0.44/0.34	497 for each	CAD
Dosenko et al. [91]	2006	Ukraine	4b/a, T786-C	-	0.20/0.21	0.34/0.29	304 for each	ACS
Jaramillo et.al. [21]	2006	Chile	Glu298Asp	0.21/0.15	-	-	184	CAD
Rios et al.(1) [57]	2007	Brazil	Glu298Asp, 4b/a	0.29/0.26	0.18/0.23	-	447 for each	CAD
Sampaio et al. [58]	2007	Brazil	Glu298Asp, 4b/a, T786-C	0.31/0.28	0.16/0.19	0.25/0.37	219 for each	PMI
Andrikopoulos et al. [59]	2008	Greece	Glu298Asp	0.33/0.31	-	-	2329	AMI
Jaramillo et al. [92]	2008	Chile	T786-C	-	-	0.24/0.22	221	CAD
Szperl et al. [60]	2008	Poland	Glu298Asp	0.32/0.27	-	-	418	CAD
Vasilakou et al. [61]	2008	Greece	Glu298Asp, 4b/a	0.27/0.30	0.19/0.15	-	370 for each	CAD
Gluba et al. [62]	2009	Poland	Glu298Asp, T786-C	0.50/0.54	-	0.36/0.38	412 for each	PCAD
Kincl et al. [105]	2009	Czech republic	4b/a	-	0.18/0.18	-	1161	CAD
Meluzin et al. [93]	2009	Czech republic	4b/a, T786-C	-	0.18/0.15	0.38/0.32	419 for each	CAD
Dafni et al. [15]	2010	Greece	Glu298Asp	0.36/0.29	-	-	422	MI
Isordia-Salas et al. [63]	2010	Mexico	Glu298Asp	0.25/0.14	-	-	360	PMI
Jaramillo et al. [39]	2010	Chile	Glu298Asp, 4b/a, T786-C	0.21/0.18	0.08/0.08	0.24/0.22	224 for each	CAD
Ragia et al. [32]	2010	Greece	Glu298Asp, T786-C	0.31/0.32	-	0.44/0.47	309	CAD
da Costa Escobar Piccoli et al. [64]	2012	Brazil	Glu298Asp, 4b/a, T786-C	0.34/0.26	0.22/0.21	0.36/0.33	241, 240 and 246 respectively for each	ACS
Zigra et al. [65]	2013	Greece	Glu298Asp, T786-C	0.32/0.31	-	0.39/0.19	210	PMI
**MIDDLE EASTERN ANCESTRY GROUP**								
Aras et al. [66]	2002	Turkey	Glu298Asp	0.33/0.28	-	-	398	CAD
Cine et al. [34]	2002	Turkey	4b/a	-	0.18/0.10	-	513	MI
Afrasyap et al. [67]	2004	Turkey	Glu298Asp	0.34/0.30	-	-	400	CAD
Berdeli et al. [68]	2005	Turkey	Glu298Asp	0.46/0.17	-	-	198	PCAD
Cam et al. [69]	2005	Turkey	Glu298Asp	0.46/0.17	-	-	198	PCAD
Matyar et al. [106]	2005	Turkey	4b/a	-	0.21/0.14	-	266	CAD
Agirbasli et al. [107]	2006	Turkey	4b/a	-	0.14/0.10	-	204	PCAD
Kerkeni et al. [70]	2006	Tunisia	Glu298Asp	0.33/0.22	-	-	220	CAD
Salimi et al. [109]	2006	Iran	4b/a	-	0.17/0.10	-	299	CAD
Tangurek et al. [94]	2006	Turkey	T786-C	-	-	0.37/0.26	211	CAD
Jemaa et al. [110]	2007	Tunisia	4b/a	-	0.22/0.14	-	560	MI
Ciftci et al. [95]	2008	Turkey	T786-C	-	-	0.33/0.19	61	CAD
Alp et al. [23]	2009	Turkey	Glu298Asp, T786-C	0.28/0.25	-	0.29/0.29	268 for each	CAD
Alkharfy et al. [71]	2010	Saudi Arabia	Glu298Asp	0.31/0.19	-	-	287	CAD
Bor-Kucukatay et al. [14]	2010	Turkey	Glu298Asp	0.25/0.18	-	-	157	CAD
Salimi et al. [16]	2010	Iran	Glu298Asp	0.32/0.23	-	-	502	CAD
Agirbasli et al. [108]	2011	Turkey	4b/a	-	0.13/0.15	-	180	PCAD
Motawi et al. [72]	2011	Egypt	Glu298Asp	0.37/0.34	-	-	150	CAD
Gad et al. [73]	2012	Egypt	Glu298Asp	0.27/0.25	-	-	205	AMI
Rahimi et al. [74]	2012	Iran	Glu298Asp	0.26/0.26	-	-	309	CAD
Salimi et al. [17]	2012	Iran	T786-C	-	-	0.33/0.20	502	CAD
Abdel Aziz et al. [75]	2013	Egypt	Glu298Asp	0.39/0.26	-	-	235	PCAD
Ekmekci et al. [111]	2013	Turkey	4b/a	-	0.20/0.09	-	120	PCAD
Kallel et al. [76]	2013	Tunisia	Glu298Asp, 4b/a, T786-C	0.26/0.26	0.22/0.14	0.37/0.35	528 for each	MI
**ASIAN ANCESTRY GROUP**								
Hibi et al. [11]	1998	Japan	Glu298Asp, 4b/a	0.09/0.09	0.12/0.11	-	583 for each	AMI
Ichihara et al. [35]	1998	Japan	4b/a	-	0.14/0.10	-	1005	MI
Odawara et al. [112]	1998	Japan	4b/a	-	013/0.08	-	164	CAD
Shimasaki et al. [10]	1998	Japan	Glu298Asp	0.11/0.07	-	-	892	MI
Nakagami et al. [113]	1999	Japan	4b/a	-	0.25/0.16	-	74	CAD
Nakayama et al. [31]	2000	Japan	T786-C	-	-	0.12/0.04	554	MI
Park et al. [36]	2000	Korea	4b/a	-	0.14/0.12	-	327	AMI
Yoon et al. [77]	2000	Korea	Glu298Asp	0.08/0.07	-	-	238	CAD
Lee et al. [114]	2001	Korea	4b/a	-	0.10/0.12	-	520	CAD
Takagi et al. [96]	2001	Japan	T786-C	-	-	0.12/0.11	4372	MI
Wang et al. [78]	2001	China	Glu298Asp	0.10/0.10	-	-	436	CAD
Hwang et al. [115]	2002	Taiwan	4b/a	-	0.11/0.10	-	219	CAD
Jo et al. [79]	2006	Korea	Glu298Asp, T786-C	0.10/0.09	-	0.16/0.11	932 for each	MI
Kim et al. [22]	2007	Korea	Glu298Asp, 4b/a, T786-C	0.09/0.09	0.13/0.09	0.13/0.09	366, 358 and 367 respectively for each	CAD
Lin et al. [80]	2008	Taiwan	Glu298Asp, 4b/a	0.42/0.22	0.14/0.11	-	198	CVD
Tamemoto et al. [81]	2008	Japan	Glu298Asp	0.18/0.06	-	-	337	CAD
Bae et al. [82]	2010	Korea	Glu298Asp, 4b/a, T786-C	0.09/0.08	0.12/0.09	0.12/0.08	388 for each	CAD
Han et al. [97]	2010	China	T786-C	-	-	0.23/0.15	622	CAD
Min et al. [83]	2010	Korea	Glu298Asp	0.11/0.04	-	-	438	CAD
**ASIAN-INDIAN ANCESTRY GROUP**								
Mathew et al. [84]	2008	India	Glu298Asp	0.15/0.12	-	-	200	CAD
Angeline et al. [13]	2010	India	Glu298Asp	0.29/0.17	-	-	200	AMI
Bhanushali et al. [85]	2010	India	Glu298Asp	0.18/0.17	-	-	200	CAD
Gururajan et al. [116]	2010	India	4b/a	-	0.28/0.14	-	206	ACS
Saini et al. [86]	2011	India	Glu298Asp	0.12/0.06	-	-	110	CAD
Rai et al. [87]	2012	India	Glu298Asp	0.21/0.17	-	-	427	CAD
Saini et al. [88]	2012	India	Glu298Asp	0.10/0.06	-	-	110	CAD
Arun Kumar et al. [89]	2013	India	Glu298Asp, T786-C	0.15/0.15	-	0.23/0.20	887 for each	MI
Narne et al. [90]	2013	India	Glu298Asp, 4b/a, T786-C	0.21/0.16	0.19/0.19	0.29/0.18	281 for each	CAD
**AFRICAN ANCESTRY GROUP**								
Hooper et al. [98]	1999	USA	4b/a	-	0.36/0.26	-	295	MI
Rao et al.(2) [104]	2005	USA	4b/a	-	0.36/0.26	-	43	CAD
Rios et al.(2) [57]	2007	Brazil	Glu298Asp, 4b/a, T786-C	0.27/0.22	0.21/0.20	0.32/0.22	268 for each	CAD

The studies were grouped according to predominant ancestry among which they were conducted.

Abbreviations used- CAD: Coronary Artery Disease; MI: Myocardial Infarction; AMI: Acute Myocardial Infarction; ACS: Acute Coronary Syndrome; PCAD: Premature CAD; PMI: Premature MI; CVD: Cardiovascular Disease

### Meta-analysis for *NOS3* Glu298Asp polymorphism

#### Pooled analysis

**Figure 2:**
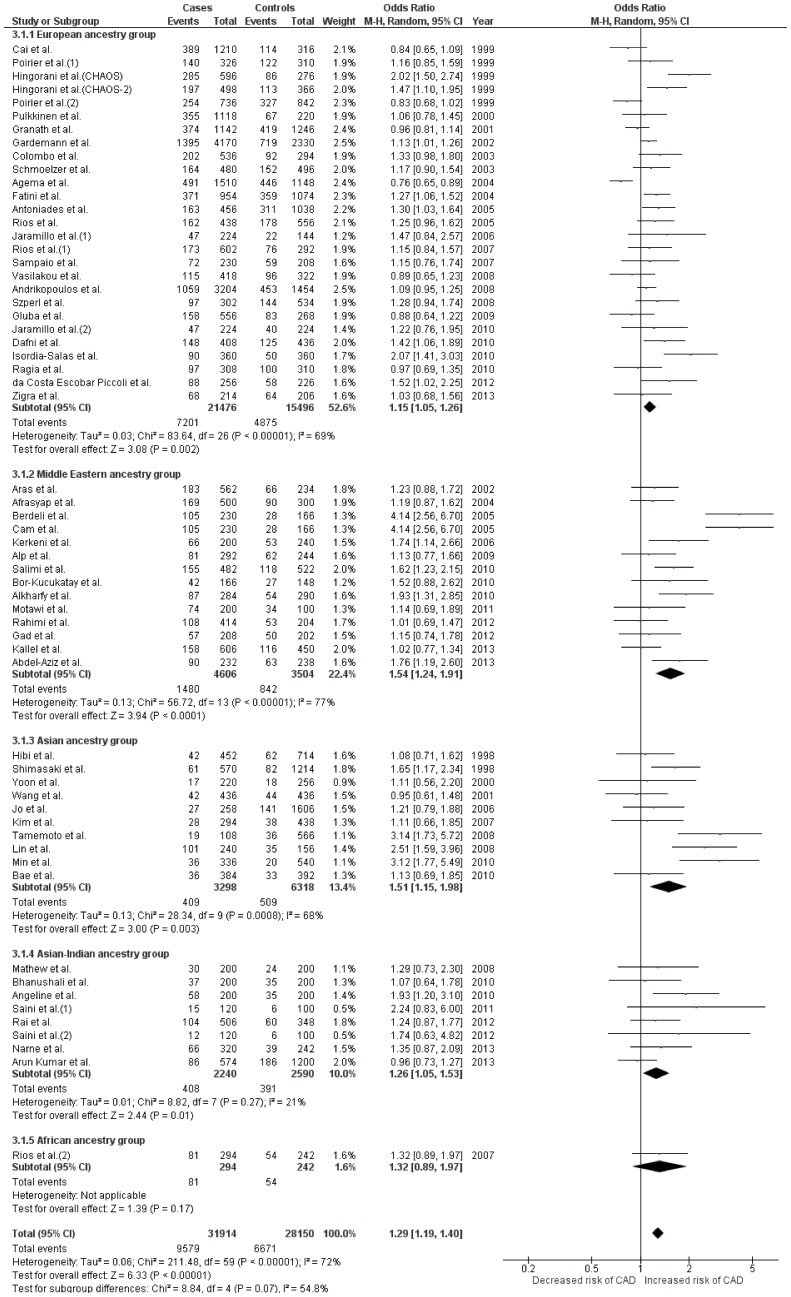
Forest plot depicting results after analyses for the allelic model *(Allele T vs. G)* of *NOS3* Glu298Asp polymorphism. Effect size estimates for all ancestral groups in this plot were obtained using random effects for analysis. Effect size using fixed effects was recalculated for Asian-Indian group which showed homogenous distribution among its included studies. Recalculated effect size estimate for Asian-Indians was, OR, 95%CI = 1.23, 1.05–1.44; Z = 2.59; P = 0.01.

A total of 60 case control studies [2], [3], [5], [10], [11], [13]–[16], [18], [20]–[23], [28], [32], [37]–[39], [53]–[90] investigating the association between Glu298Asp polymorphism and CAD, with a total of 15,957 CAD patients and 14,075 normal controls were included in the pooled analysis (All ancestries combined). (**Tables 1** **and** **2**) This analysis, using three different genetic models showed moderate to high heterogeneity (*I^2^* range = 58–72% and P_Q_<0.00001 for all comparisons). (**Table 2**) All three genetic models for Glu298Asp also showed significant association with CAD (OR range = 1.28–1.52, Z value range = 5.14–6.33 and P<0.00001 for all comparisons). (**Table 3** **and** **Figures 2** **, S1 and S2**)

**Table 2:** Basic characteristics of the studied groups classified according to predominant ancestries.

	Heterogeneity (*I^2^*; P_Q_)			Number of Studies	Cases (n)/Controls (n)	Total sample
	*Dominant genetic model* *	*Recessive genetic model* **	*Allelic genetic model* ***			
**Glu298Asp polymorphism**						
All ancestries combined	65%; <0.00001	58%; <0.00001	72%; <0.00001	60	15,957/14,075	30,032
European ancestry group	52%; 0.0008	63%; <0.00001	69%; <0.00001	27	10,738/7,748	18,486
Middle Eastern ancestry group	64%; 0.0005	65%; 0.0005	77%; <0.00001	14	2,303/1,752	4,055
Asian ancestry group	78%; <0.00001	33%; 0.16	68%; 0.0008	10	1,649/3,159	4,808
Asian-Indian ancestry group	18%; 0.28	23%; 0.26	21%; 0.27	8	1,120/1,295	2,415
African ancestry group	-	-	-	1	147/121	268
**T786-C polymorphism**						
All ancestries combined	66%; <0.00001	35%; 0.03	69%; <0.00001	29	7,043/10,409	17,452
European ancestry group	71%; <0.00001	38%; 0.07	72%; <0.00001	15	3,977/3,234	7,211
Middle Eastern ancestry group	50%; 0.09	68%; 0.01	70%; 0.009	5	879/691	1,570
Asian ancestry group	67%; 0.009	0%; 0.69	68%; 0.009	6	1,593/5,642	7,235
Asian-Indian ancestry group	50%; 0.16	70%; 0.07	69%; 0.07	2	447/721	1,168
African ancestry group	-	-	-	1	147/121	268
**4b/a polymorphism**						
All ancestries combined	60%; <0.00001	17%; 0.17	61%; <0.00001	43	12,477/9,274	21,751
European ancestry group	51%; 0.005	0%; 0.72	47%; 0.01	20	8,779/5,373	14,152
Middle Eastern ancestry group	0%; 0.83	0%; 0.66	0%; 0.80	8	1,356/1,314	2,670
Asian ancestry group	28%; 0.18	0%; 0.72	0%; 0.50	10	1,797/2,039	3,836
Asian-Indian ancestry group	87%; 0.005	0%; 0.65	87%; 0.005	2	266/221	487
African ancestry group	33%; 0.22	0%; 0.58	0%; 0.37	3	279/327	606

Abbreviations: P_Q_: Cochran's Q statistics; *I^2^*: Higgin's *I^2^* statistics.

*Dominant genetic model for Glu298Asp: *TT+GT vs. GG;* for T786-C: *CC+CT vs. TT;* for 4b/a: *4a4a+4a4b vs. 4b4b.*

**Recessive genetic model for Glu298Asp: *TT vs. GG+GT;* for T786-C: *CC vs. TT+CT;* for 4b/a: *4a4a vs. 4a4b+4b4b.*

***Allelic genetic model for Glu298Asp: *Allele T vs. G;* for T786-C: *Allele C vs. T;* for 4b/a: *Allele 4a vs. 4b.*

Notes: Heterogeneity was tested among groups of studies using Higgin's *I^2^* statistics and Cochran's Q statistics (P_Q_). Group showing *I^2^*>50% and P_Q_<0.01 were considered heterogeneous, others were considered homogenous.

**Table 3:** Meta-analysis results for *NOS3* gene polymorphisms.

	*Dominant genetic model* *		*Recessive genetic model* **		*Allelic genetic model* ***	
	OR, 95% CI	Z; P value	OR, 95% CI	Z; P value	OR, 95% CI	Z; P value
**Glu298Asp polymorphism**						
All ancestries combined	1.28, 1.17-1.40	5.34; <0.00001	1.52, 1.30-1.79	5.14; <0.00001	1.29, 1.19-1.40	6.33; <0.00001
European ancestry group	1.11, 1.01–1.22	2.10; 0.04	1.36, 1.14–1.63	3.37; 0.0007	1.15, 1.05–1.26	3.08; 0.002
Middle Eastern ancestry group	1.55, 1.25–1.93	3.94; <0.0001	1.87, 1.23–2.85	2.92; 0.004	1.54, 1.24–1.91	3.94; <0.0001
Asian ancestry group	1.60, 1.12–2.29	2.56; 0.01	2.22, 1.21–4.08^F^	2.57; 0.01^F^	1.51, 1.15–1.98	3.00; 0.003
Asian-Indian ancestry group	1.21, 1.01–1.45^F^	2.07; 0.04^F^	1.85, 1.12–3.04^F^	2.42; 0.02^F^	1.23, 1.05–1.44^F^	2.59; 0.01^F^
African ancestry group	1.22, 0.75–1.99	0.81; 0.42	2.84, 0.90–8.94	1.78; 0.07	1.32, 0.89–1.97	1.39; 0.17
**T786-C polymorphism**						
All ancestries combined	1.42, 1.25–1.62	5.24; <0.00001	1.36, 1.20–1.53^F^	4.97; <0.00001^F^	1.34, 1.20–1.49	5.32; <0.00001
European ancestry group	1.25, 1.02–1.52	2.19; 0.03	1.32, 1.15–1.52^F^	3.96; <0.0001^F^	1.21, 1.05–1.40	2.56; 0.01
Middle Eastern ancestry group	1.59, 1.16–2.18	2.91; 0.004	1.35, 0.69–2.65	0.87; 0.38	1.43, 1.04–1.97	2.20; 0.03
Asian ancestry group	1.67, 1.26–2.23	3.51; 0.0004	1.90, 1.15–3.14^F^	2.50; 0.01^F^	1.61, 1.24–2.10	3.56; 0.0004
Asian-Indian ancestry group	1.53, 1.04–2.26	2.16; 0.03	1.73, 0.38–7.95	0.71; 0.48	1.44, 0.95–2.18	1.71; 0.09
African ancestry group	1.91, 1.17–3.12	2.58; 0.010	1.85, 0.77–4.44	1.37; 0.17	1.70, 1.15–2.52	2.67; 0.008
**4b/a polymorphism**						
All ancestries combined	1.19, 1.07–1.32	3.11; 0.002	1.41, 1.19–1.67^F^	3.99; <0.0001^F^	1.20, 1.09–1.32	3.77; 0.0002
European ancestry group	1.00, 0.89–1.13	0.06; 0.95	1.03, 0.83–1.26^F^	0.24; 0.81^F^	0.99, 0.93–1.06^F^	0.19; 0.85^F^
Middle Eastern ancestry group	1.73, 1.46–2.05^F^	6.28; <0.00001^F^	3.02, 1.90–4.81^F^	4.65; <0.00001^F^	1.71, 1.47–1.98^F^	7.09; <0.00001^F^
Asian ancestry group	1.22, 1.04–1.42^F^	2.45; 0.01^F^	2.48, 1.38–4.47^F^	3.03; 0.002^F^	1.25, 1.08–1.44^F^	3.06; 0.002^F^
Asian-Indian ancestry group	1.57, 0.53–4.64	0.82; 0.41	2.63, 1.02–6.82^F^	1.99; 0.05^F^	1.56, 0.62–3.94	0.94; 0.35
African ancestry group	1.37, 0.99–1.90^F^	1.87; 0.06^F^	2.02, 1.04–3.91^F^	2.07; 0.04^F^	1.37, 1.05–1.78^F^	2.34; 0.02^F^

Abbreviations: OR, 95% CI: Odds Ratio with its 95% Confidence Interval; **^F^**: Results derived using Fixed effects for analysis. Random effects were used for all other calculations.

*Dominant genetic model for Glu298Asp: *TT+GT vs. GG;* for T786-C: *CC+CT vs. TT;* for 4b/a: *4a4a+4a4b vs. 4b4b.*

**Recessive genetic model for Glu298Asp: *TT vs. GG+GT;* for T786-C: *CC vs. TT+CT;* for 4b/a: *4a4a vs. 4a4b+4b4b.*

***Allelic genetic model for Glu298Asp: *Allele T vs. G;* for T786-C: *Allele C vs. T;* for 4b/a: *Allele 4a vs. 4b.*

#### Subgroup analysis

Among subgroups for Glu298Asp, 17 studies belonged to the European, [2], [3], [5], [15], [18], [20], [21], [28], [32], [37]–[39], [53]–[65] 14 to the Middle Eastern, [14], [16], [23], [66]–[76] 10 to the Asian, [10], [11], [22], [77]–[83] 8 to the Asian-Indian [13], [84]–[90] and 1 to the African [57] group. (**Tables 1** **and** **2**) The Asian-Indian subgroup in all its genetic models showed low heterogeneity (*I^2^* range = 18–23% and P_Q_ range = 0.26–0.28). All other ancestral groups (apart from African which had only 1 study) showed moderate to high heterogeneity (*I^2^* range = 33–78% and P_Q_ range = 0.16 to <0.00001). (**Table 2**) Appropriate effects were thus used for further analysis. Four out of 5 ancestral groups (except Africans) showed significant association with CAD in all three genetic models. The Middle Eastern group showed highest levels of association amongst the studied groups (Z value range = 2.92–3.94 and P≤0.004 for all comparisons), followed by European group (Z value range = 2.10–3.37 and P≤0.04 for all comparisons). (**Table 3** **and** **Figures 2** **, S1 and S2**)

### Meta-analysis for *NOS3* T786-C polymorphism

#### Pooled analysis

**Figure 3:**
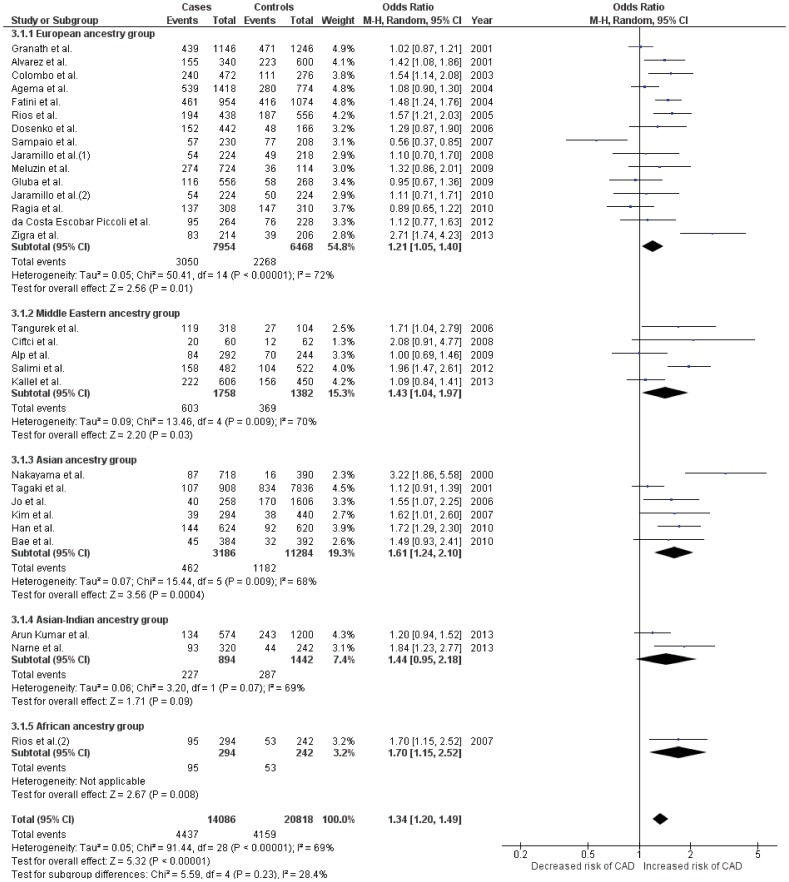
Forest plot depicting results after analyses for the allelic model *(Allele C vs. T)* of *NOS3* T786-C polymorphism. Effect size estimates for all ancestral groups in this plot were obtained using random effects for analysis.

Twenty nine case-control studies [5], [17], [22], [23], [27], [28], [31], [32], [37], [39], [54], [55], [57], [58], [62], [64], [65], [76], [79], [82], [89]–[97] with 7,043 CAD patients and 10,409 controls, investigating the association between T786-C polymorphism and CAD were included in the pooled analysis.(**Tables 1** **and** **2**) Moderate heterogeneity was seen across all genetic models (*I^2^* range = 35–69%, P_Q_ range = 0.03 to <0.00001). (**Table 2**) All genetic models also showed significantly associated with CAD showing ORs in the range of 1.34–1.42, Z value ranging from 4.97–5.32 and P values of <0.00001 (for all comparisons). (**Table 3** **and** **Figures 3** **, S3 and S4**)

#### Subgroup analysis

Among the ancestral subgroups, 15 studies constituted the European group, [5], [27], [28], [32], [37], [39], [54], [55], [58], [62], [64], [65], [91]–[93] while 5,6,2 and 1 study respectively constituted Middle Eastern, [17], [23], [76], [94], [95] Asian, [22], [31], [79], [82], [96], [97] Asian-Indian [89], [90] and African [57] group. (**Tables 1** **and** **2**) Moderate to high degree of heterogeneity was seen in almost all ancestral groups (except Africans, which had only 1 study), and in amongst almost all comparisons, barring the recessive model amongst the Asians which was rather homogenous (*I^2^* range = 50–72%, P_Q_ range = 0.16 to <0.00001). (**Table 2**) All ancestral subgroups showed significant association in dominant genetic model, similarly all (except Asian-Indians) showed significant association in the allelic model. On the other hand, only Europeans and Asians showed significant association in recessive model. If we disregard the Africans which had only 1 study in its subgroup, Asians showed the highest degree of association consistently in all three models (OR range = 1.61–1.90, Z value range = 2.50–3.56 and P value range = 0.01–0.0004), followed by the Europeans (OR range = 1.21–1.32, Z value range = 2.19–3.96 and P value range = 0.03 to <0.0001). (**Table 3** **and** **Figures 3** **S3 and S4**)

### Meta-analysis *4b/a (27bp VNTR)* polymorphism

#### Pooled analysis

**Figure 4:**
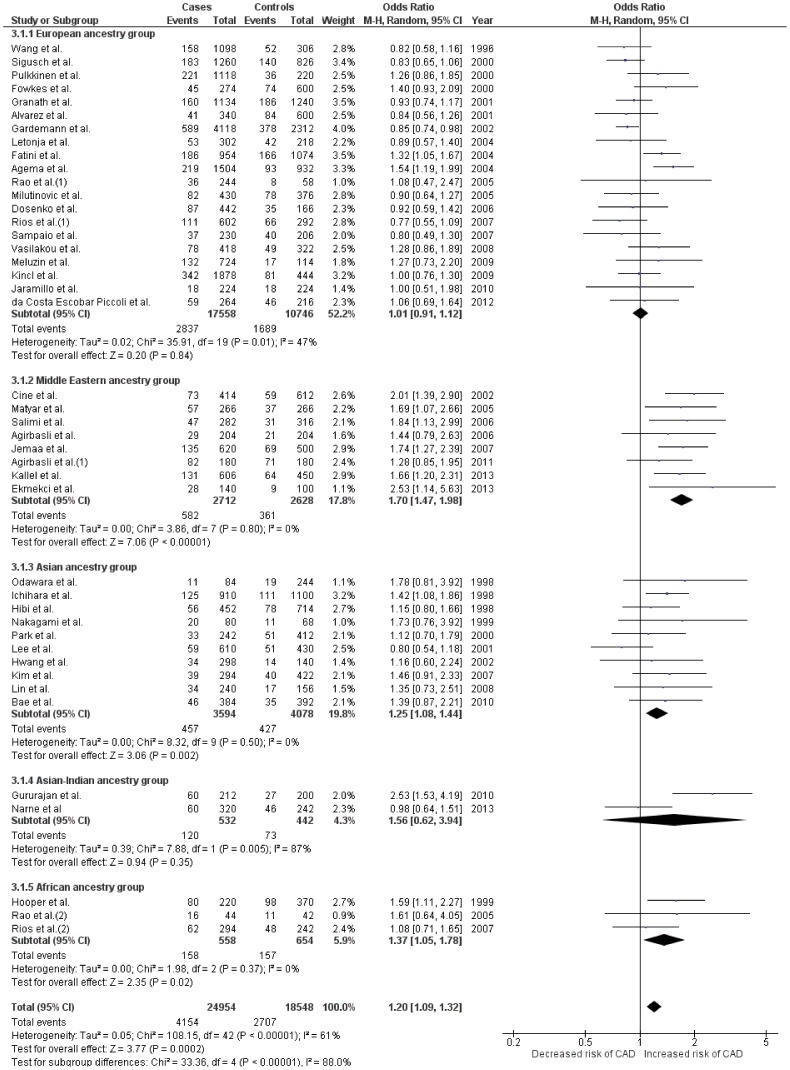
Forest plot depicting results after analyses for the allelic model *(Allele 4a vs. 4b)* of *NOS3* 4b/a VNTR polymorphism. Effect size estimates for all ancestral groups in this plot were obtained using random effects for analysis. Effect sizes using fixed effects were recalculated for European, Middle Eastern, Asian and African groups which showed homogenous distribution among its included studies. Recalculated effect size estimates were, OR, 95%CI = 0.99, 0.93–1.06; Z = 0.19; P = 0.85 for Europeans; OR, 95%CI = 1.71, 1.47–1.98; Z = 7.09; P<0.00001 for Middle Easterners; OR, 95%CI = 1.25, 1.08–1.44; Z = 3.06; P = 0.002 for Asians and OR, 95%CI = 1.37, 1.05–1.78; Z = 2.34; P = 0.02 for Africans.

Forty three case-control studies, [2], [11], [22], [27], [33]–[37], [39], [54], [55], [57], [58], [61], [64], [76], [80], [82], [90], [91], [93], [98]–[116] with 12,477 CAD patients and 9,274 controls analysing the association of 27bp b/a VNTR polymorphism and CAD were included in the pooled analysis. (**Table 2**) Low to moderate heterogeneity was seen among the three genetic models (*I^2^* = 17–61%, P_Q_ = 0.17 to <0.00001). (**Table 2**) However, all three genetic models consistently showed significant association with CAD (OR range = 1.19–1.41, Z value range = 3.11–3.99, P≤0.002 for all comparisons). (**Table 3** **and** **Figures 4** **, S5 and S6**)

#### Subgroup analysis

A total of 20 studies from European, [2], [27], [33], [37], [39], [54], [55], [57], [58], [61], [64], [91], [93], [99]–[105] 8 from Middle Eastern, [34], [76], [106]–[111] 10 from Asian, [11], [22], [35], [36], [80], [82], [112]–[115] 2 from Asian-Indian [90], [116] and 3 from African [57], [98], [104] group were included for subgroup analysis. (**Table 2**) Middle Eastern, Asian and African groups showed considerably low heterogeneity among the three genetic models (*I^2^ range* = 0–33%; P_Q_ range = 0.18–0.83), while European and Asian-Indian groups showed very low to very high heterogeneity (*I^2^ range* = 0–87%; P_Q_ range = 0.72–0.005). (**Table 2**) The Middle Eastern and Asian groups showed significant association in all three models. It is however noteworthy that the Middle Eastern subgroup showed stronger association (OR range = 1.71–3.02, Z value range = 4.65–7.09, P<0.0001 for all comparisons) than the Asian subgroup (OR range = 1.22–2.48, Z value range = 2.45–3.06, P≤0.01 for all comparisons) consistently in all three models. African group showed moderate association limited only to recessive and allelic models (OR range = 1.37–2.02, Z value range = 2.07–2.34, P≤0.02), whereas Asian-Indian group showed marginal association in only the recessive genetic model (OR = 2.63, Z value = 1.99, P value = 0.05). The European group however showed no association in any of the three genetic models (P>0.05). (**Table 3** **and** **Figures 4** **, S5 and S6**)

### Source of heterogeneity

Higher degree of heterogeneity was seen amongst almost all ancestral groups (except Asian-Indians) in all comparisons for Glu298Asp polymorphism. All groups were fairly heterogeneous in all comparisons for T786-C polymorphism. Fairly low heterogeneity was seen amongst Middle Eastern and Asian groups in all comparisons for 4b/a polymorphism. (**Table 2**) Environmental factors like diet, exercise etc. could be the probable reasons behind seen heterogeneity among the groups. We did all we could by classifying studies into five broad subgroups, based on predominant ancestry, in order to determine sources of heterogeneity. Further classification would have only made the samples in each subgroup smaller and their results impotent. (**Table 2**)

### Potential publication Bias

Potential publication bias among groups of studies for each SNP was assessed by two different statistical tests, viz. Egger's plots [51] and Begg's funnel plots. [50] The recessive genetic model showed least heterogeneity for all three SNPs among all ethnic groups (as seen in **Table 2**), its data was thus selected to generate Egger's plots. No asymmetry in plots was seen for Glu298Asp or 4b/a for the pooled group or any of its ancestral subgroups. Egger's P values for Glu298Asp were 0.56, 0.11, 0.08, 0.42 and 0.56 respectively for pooled and European, Middle Eastern, Asian and Asian Indian subgroups. Similarly Egger's P values for 4b/a were 0.79, 0.15, 0.85, 0.92, 0.88 for pooled and European, Middle Eastern, Asian and African subgroups. Owing to less number of studies (n<3), no P value from Egger's test was generated in African subgroup of Glu298Asp and Asian-Indian subgroup of 4/a polymorphism. In case of T786-C, Egger's plot asymmetry was however seen for pooled (P = 0.002) and European (P = 0.004) subgroup. Rest of the two subgroups showed either symmetry in their plots (P = 0.71 and 0.91 for Middle Eastern and Asian groups respectively) or were their p values was incalculable (i.e. for Asian-Indian and African groups).

**Figure 5:**
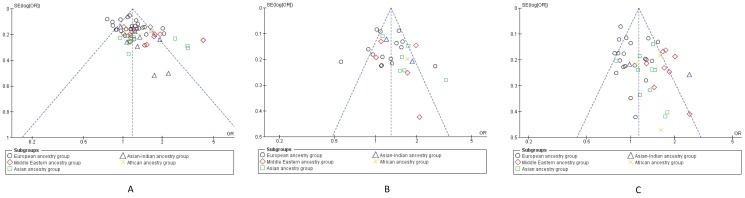
Begg's funnel plots for testing publication bias in Allelic comparisons. Each point in each figure represents OR of a study plotted against the standard error (SE) its OR. Different indicators of the studies belonging to each ancestral group are used in these plots. **Figure 5A**: *Allele T vs. G* for Glu298Asp; **Figure 5B**: *Allele C vs. T* for T786-C; **Figure 5C**: *Allele 4a vs. 4b* for 4b/a.

Begg's funnel plots, [50] were also generated to assess potential publication bias for each SNP in their three genetic models. Funnel plots are provided for pooled analysis obtained in allelic comparisons for all three studied polymorphisms. (**Figure 5-A, B, C**) Plots for dominant and recessive models for all three SNPs are also provided as (**Figures S7-A, B; S8-A, B; S9-A, B**). Each point in the figure represents OR of a study plotted against standard error (SE) of OR of that particular study. Different indicators of the studies belonging to each ancestry are used in these plots where they are seen to be generally contained within the inverted cone, indicating no significant publication bias, at least in the pooled analyses.

### Sensitivity Analysis

Sensitivity analysis was performed in each study group in every genetic model, where we excluded studies one after another and conducted the analysis after each omission. The results in none of the groups and studied genetic models altered substantially to change the results from no association to significant association or the other way around (in groups of ≥5 studies). This indicates that the present meta-analysis is robust in nature.

## Discussion

The present meta-analysis and structured systematic review is the most comprehensive till date and comprises of published studies which investigated the association of three most common *NOS3* gene polymorphisms, (i.e. Glu298Asp, 4b/a and T786-C) with CAD.

All comparisons in the pooled analysis for all three SNPs indicated significant association with CAD. The pooled results of Glu298Asp suggested that the presence of even a single T allele in a genotype effects in an increased risk of CAD. The risk of CAD is even higher if the genotype contains more than 1, T allele. This association is evident by the obtained pooled OR in allelic comparison (OR = 1.29, Z value = 6.33 and p<0.00001). (**Table 3** **and** **Figure 2**) The same pattern of association is seen for almost all the ancestral groups (except Africans), where generally higher ORs are seen in recessive models as compared to their dominant models. (**Table 3** **and Figures S1 and S2**) Consistent to the seen pattern, the T allele also showed a significant association with CAD in almost all ancestral groups, barring the Africans which had only 1 study. (**Table 3** **and** **Figure 2**) In the case of T786-C polymorphism, our pooled as well as subgroup results also suggested an increased risk in genotypes carrying the minor (C) allele. All three genetic models showed significant association in the pooled analysis. Significant association was also seen in all ancestral subgroups in at least two out of three genetic models. As in the case of Glu298Asp, the risk increased in genotypes of T786-C, which were homozygous to the C allele. However, contrary to the trends seen in case of Glu298Asp and 4b/a, we observed that for T786-C, Asians followed by Europeans showed highest degree of associations across all three genetic models. (**Table 3** **and** **Figure 3**) The presence of 4a allele of 4b/a polymorphism resulted in an increased risk for CAD in the pooled analysis (OR = 1.20) and specifically among two ancestral groups viz. Middle Eastern (OR = 1.71) and Asian (OR = 1.25). (**Table 3** **and** **Figure 4**) Here too, genotypes carrying >1, 4a alleles showed notably higher risk in pooled analysis as well as in the Middle Eastern and Asian subgroups. The pooled as well as the Middle Eastern and Asian subgroups also showed significant association in the allelic model. As far as the 4b/a polymorphism is concerned, Africans showed significant association in recessive and allelic model, while Asian-Indians showed a hint of association in the recessive model. (**Table 3** **and** **Figures 4** **, S5 and S6**)

Our pooled analysis showed significant associations of all three selected SNPs with CAD in every employed genetic model. Our results are in contrast to the previously published meta-analysis (for all three selected SNPs), [40] which was not able to deduce any significant associations for T786-C and 4b/a and only showed significant association for Glu298Asp under dominant genetic model. One reason for the difference in the results of the present study and the study by Li et al., [40] can be the sheer number of studies included. Since the year 2010 when the study by Li et al. [40] was published, a number of studies came out in the public domain among which several of them reported statistically significant associations. This previous meta-analysis [40] included a much lesser number of studies, thus making the present study a more comprehensive one (56 vs. 132 studies in previous vs. present study respectively). Several other recent meta-analyses which assessed either one of the three *NOS3* SNPs are also now into public domain. [41]–[44] The pooled analysis for Glu298Asp in the meta-analysis by Tian et al., [43] reported significant associations in dominant (OR = 1.17), recessive (OR = 1.59) and allelic (OR = 1.26) models which are somewhat comparable to those deduced in our study. In the meta-analysis by Tian et al. [43], the Asian subgroup for Glu298Asp showed significant association to CHD in all employed genetic models, the effect sizes of which are similar to those deduced for the Asian subgroup in the present study. There is also a meta-analysis by Zhang et al., [41] who reported significant association (with OR = 1.52), in allelic comparisons of Glu298Asp among Asians, the effect size of which is similar to that deduced in our study (OR = 1.51). We also obtained similar results for Asian subgroup with another previously published meta-analysis for T786-C [42]. In fact our study can be considered as an improvement on all the previously published meta-analyses on polymorphic forms of *NOS3* gene and CAD, [40]–[44] not only in the terms of higher number of studies included but also in terms of better classification of ancestral groups and assessment of their individual risk. This allowed us to unravel the degree of associations among several other important ancestral groups such as Middle Easterners and Asian-Indians apart from Europeans and Asians which were already amply investigated by several previously published meta-analyses.

There are basically two major merits of the present study which makes it superior to all previously published meta-analyses on this subject. [40]–[44] Firstly, our study is the most comprehensive till date and we ended up including most number of individual studies which transcribed into the largest sample size ever studied for each of the three *NOS3* SNPs. Secondly, our study had strict inclusion/exclusion criteria, and we excluded studies with surrogate endpoints, and omitted studies in which controls did not conform to HWE proportions (most of which were included in previously published meta-analyses). This methodology ensured that we eliminated studies with potentially biased/inaccurate results, thus possibly presenting a real picture.

The biochemical basis of the association of CAD with various *NOS3* gene polymorphisms has been studied in detail by several researchers. Most schools of thought suggest that all these SNPs of *NOS3* gene viz. Glu298Asp, T786-C and 4b/a 27bp VNTR via various mechanisms affect the serum nitrite/nitrate (NO*_x_*) concentrations in the human body. Over a period of time this imbalance in NO*_x_* concentrations results in “endothelial dysfunction” which finally blooms into an overt form of CAD. It is plausible that this degree of association with CAD and the resulting NO*_x_* concentrations are affected by gene-gene, gene-environment interactions. Compensatory mechanism of the human body against CAD may also increase NO secretion from the vascular endothelium during sudden reperfusion after acute coronary syndromes. [117], [118] Therefore it remains unclear whether the increase in NO levels is a “cause” or an “effect” of acute coronary syndromes. Clearly the association between these SNPs and CAD, probably also has different biochemical basis rather than solely brought about by variations in NO*_x_* concentrations.

Positive association of Glu298Asp with CAD/MI has been reported over the years in several published studies. We found that along with a significant association for all three genetic models in our pooled analysis, this SNP seen to be associated with CAD separately among all ancestral groups, with the highest degree of association seen amongst the Middle Easterners.(**Table 3** **and** **Figures 2** **, S1 and S2**) The reason of this differential risk of CAD seen among different ancestral groups carrying this polymorphism is unknown and should be investigated upon. We presume that this difference could had been brought about by the variance in Asp allele frequency (range = 0.04-0.39), as well as several known and unknown gene-gene and gene-environment interactions. The biochemical basis of the association of CAD with Asp allele carriers has although been extensively studied upon however a clear consensus is still lacking. Some studies have reported an association of this SNP with intracellular NO concentration. [12] The mechanism by which the Asp allele possibly reduces NO bioavailability in the human body has also been hypothesized. It has been suggested that the Asp298 is subjected to selective proteolytic cleavage in the endothelial cells and vascular tissues. [119], [120] The resultant cleaved fragments especially among the homozygous mutants of this SNP are expected to lack NO synthase activity [13] thus reducing bioavailability of NO in the body. [119], [120] Contrary to this notion several studies [13], [14], [67] have reported lack of association of plasma NO*_x_* concentrations with any of the genotypes or alleles for this SNP. Also, since these associations are often masked, as it is affected by several physiological and pathological conditions, the real effect of *NOS3* gene on plasma NO*_x_* levels is hard to determine. [14] As already discussed genotypes carrying Asp alleles impart greater RBC aggregability. [14] Genotypes having Asp alleles have also been shown to have (i) a reduced blood pressure fall after exercise training, [121] (ii) an enhanced systemic presser response to phenylephrine [12] (iii) lower basal blood flow and reduced vasodilation to adenosine in coronary arteries [122] and (iv) a reduced flow-mediated dilatation of the brachial artery, [123] making them more prone to suffer from coronary events. It is possible that these effects coupled with possible modulation in bioavailability of NO in presence of interactions with other genes and environmental factors may accelerate the process of endothelial dysfunction and subsequent emergence of overt CAD among Asp variants of the *NOS3* gene.

The T786-C gene variant of *NOS3* has also been implicated over the years with CAD occurrence by several investigators. Along with a significant association among all three genetic models in our pooled analysis, Asians seemed to have strongest association with CAD than other ancestral groups. (**Table 3** **,** **Figure 3** **, S3 and S4**) The reason behind this dissimilar degree of association among these ancestral groups is unknown. More in depth studies are warranted to answer this pertinent question. In vitro reporter gene assays performed by Nakayama et al., [24] somewhat explained the possible functional effect of this SNP. They demonstrated that T786-C mutation reduces the promoter activity by as high as 50%, suggesting that in several C allele carriers the L-arginine/NO pathway does not function properly, reducing NO concentrations and causing endothelial dysfunction. [24] Other studies have also shown lower *NOS3* mRNA and serum NO*_x_* levels in genotypes with ≥1, 786C alleles, [26] although contradictory results have also been published. [124] Apart from lower NOx concentrations [26], synergistic effect of *NOS3* Glu298Asp and T786-C polymorphism has also been reported to be the cause of increased CAD risk among carriers of this SNP. [5]

Clear scientific evidence is lacking which can explain the seen association of *NOS3* 4b/a VNTR polymorphism with CAD. In contrast to the previous studies, [40], [44] the pooled results of our study yielded significant association amongst all three genetic models. Highest degree of association among the Middle Eastern group was seen in all three genetic models. (**Table 2** **and** **Figures 4** **, S5 and S6**) The precise reason of this heightened risk amongst subjects of Middle Eastern ancestry is unknown and can only be speculated upon. Here again the influence of environmental factors such as diet and exercise may well be associated with the increased risk. Conflicting reports between the intron-4 variant and NO pathway activity are there in the public domain. Genotypes carrying ≥1 mutant alleles have been shown to have lower plasma NO levels and reduced protein expression, [125], [126] although some studies have demonstrated otherwise. [77], [124], [127] As this polymorphism is located in the intron region of the gene, even if it is not functional it might act as a risk factor and act in linkage disequilibrium with other regulatory regions and functional gene variants of the *NOS3* gene. [124]

We believe that the present study has successfully answered the research question that it aimed at the outset and is statistically powered enough to the stand by the results it obtained. Although, there is a scope for limitations in every meta-analysis of genetic association studies and ours was no exception either. Firstly, we believe that there was under representation of the South-Asian region, and we found no published data on the subject from several South-Asian countries like Bangladesh, Sri Lanka and Pakistan etc., which have a sizable population inflicted with CAD. Secondly possibility of errors in genotyping, presence of selection bias and risk of inadequate sample size among different studies cannot be ruled out. It is also noteworthy that in case of polygenic diseases like CAD, an association study cannot test causality, it can merely measure statistical associations. Also, during meta-analysis of association studies we cannot ensure interference of linkage disequilibrium of the selected SNP with another closely linked site and its extent of effect on the overall association. Even after using two statistical tools, we could not detect any hint of publication bias amongst our study groups, at least for Glu298Asp and 4b/a polymorphisms. However, Egger's plot asymmetry was observed, with significant p values for pooled and European subgroup of T786-C polymorphism. Since the accuracy of Egger's test is debatable, and this asymmetry in the aforementioned plots could have possibly been due to factors like inter-study variations or small study effects among European subgroup for T786-C. Even then, the role of existing publication bias cannot be completely ruled out. This also qualifies to be listed as another small limitation in the present study.

## Conclusions

In conclusion, since the criteria for inclusion and exclusion of studies are critical parts of a meta-analysis and can substantially affect results, we find that the present study is the most comprehensive till date and is systematically planned to funnel out erroneous studies. We thus feel that our study projects the real picture, and confirms the association of the three common *NOS3* gene polymorphisms i.e. Glu298Asp, T786-C and 4a/b VNTR with CAD transcending all ancestries worldwide. Results amongst different ancestral subgroups were rather illuminating. Amongst the studied *NOS3* SNPs, both Glu298Asp and 4b/a showed strongest association among the Middle Eastern subgroup. On the other hand, T786-C and its minor allele seem to carry the highest risk for CAD among subjects of Asian ancestry. Our study significantly improves the understanding of the relation between this gene and CAD and its association amongst subjects of all major ancestries. These findings should thenceforth be assessed clinically for its implications in prevention and treatment strategies of CAD.

## Supporting Information

Figure S1 **Forest plot depicting results of meta-analysis of studies reporting** ***NOS3*** **Glu298Asp polymorphism assessed under dominant** ***(TT+GT vs. GG)*** **genetic model.** Effect size estimates for all ancestral groups in this plot were obtained using random effects for analysis. Effect sizes using fixed effects were recalculated for Asian-Indian group which showed homogenous distribution among its included studies. Recalculated effect size estimate for Asian-Indians was, OR, 95%CI = 1.21, 1.01–1.45; Z = 2.07; P = 0.04.
(TIF)

Figure S2 **Forest plot depicting results of meta-analysis of studies reporting** ***NOS3*** **Glu298Asp polymorphism assessed under recessive** ***(TT vs. GG+GT)*** **genetic model.** Effect size estimates for all ancestral groups in this plot were obtained using random effects for analysis. Effect sizes using fixed effects were recalculated for Asian and Asian-Indian groups which showed homogenous distribution among its included studies. Recalculated effect size estimates were, OR, 95%CI = 2.22, 1.21–4.08; Z = 2.57; P = 0.01 and OR, 95%CI = 1.85, 1.12–3.04; Z = 2.42; P = 0.02 for Asians and Asian-Indians respectively.
(TIF)

Figure S3 **Forest plot depicting results of meta-analysis of studies reporting** ***NOS3*** **T786-C polymorphism assessed under dominant** ***(CC+CT vs. TT)*** **genetic model.** Effect size estimates for all ancestral groups in this plot were obtained using random effects for analysis.
(TIF)

Figure S4 **Forest plot depicting results of meta-analysis of studies reporting** ***NOS3*** **T786-C polymorphism assessed under recessive** ***(CC vs. TT+CT)*** **genetic model.** Effect size estimates for all ancestral groups in this plot were obtained using fixed effects for analysis. Effect sizes using random effects were recalculated for three ancestral subgroups viz. Middle Eastern, Asian-Indian and African, which showed homogenous distribution among its included studies. Recalculated effect size estimates were, OR, 95%CI = 1.35, 0.69-2.65; Z = 0.87; P = 0.38 for Middle Easterners; OR, 95%CI = 1.73, 0.38–7.95; Z = 0.71; P = 0.48 for Asian-Indians and OR, 95%CI = 1.85, 0.77–4.44; Z = 1.37; P = 0.17 for Africans.
(TIF)

Figure S5 **Forest plot depicting results of meta-analysis of studies reporting** ***NOS3*** **4b/a VNTR polymorphism assessed under dominant** ***(4a4a+4a4b vs. 4b4b)*** **genetic model.** Effect size estimates for all ancestral groups in this plot were obtained using random effects for analysis. Effect sizes using fixed effects were recalculated for Middle Eastern and Asian groups which showed homogenous distribution among its included studies. Recalculated effect size estimates were, OR, 95%CI = 1.73, 1.46–2.05; Z = 6.28; P = <0.00001 and OR, 95%CI = 1.22, 1.04–1.42; Z = 2.45; P = 0.01 for Middle Easterners and Asians respectively.
(TIF)

Figure S6 **Forest plots depicting results of meta-analysis of studies reporting** ***NOS3*** **4b/a VNTR polymorphism assessed under recessive** ***(4a4a vs. 4a4b+4b4b)*** **genetic model.** Since all the ancestral groups showed homogenous distribution among its included studies, effect size estimates for all groups in this plot were obtained using fixed effects for analysis.
(TIF)

Figure S7 **Begg's funnel plots for testing publication bias in comparisons under different genetic models for** ***NOS3*** **Glu298Asp polymorphism.** Each point in each figure represents OR of a study plotted against the standard error (SE) its OR. Different indicators of the studies belonging to each ancestral group are used in these plots. **Figure S7A**: Begg's Plot for comparisons under dominant genetic model *(TT+GT vs. GG)*; **Figure S7B**: Begg's Plot for comparisons under recessive genetic model *(TT vs. GG+GT)*.
(TIF)

Figure S8 **Begg's funnel plots for testing publication bias in comparisons under different genetic models for** ***NOS3*** **T786-C polymorphism.** Each point in each figure represents OR of a study plotted against the standard error (SE) its OR. Different indicators of the studies belonging to each ancestral group are used in these plots. **Figure S8A**: Begg's Plot for comparisons under dominant genetic model *(CC+CT vs. TT)*; **Figure S8B**: Begg's Plot for comparisons under recessive genetic model *(CC vs. TT+CT)*.
(TIF)

Figure S9 **Begg's funnel plots for testing publication bias in comparisons under different genetic models for** ***NOS3*** **4b/a VNTR gene polymorphism.** Each point in each figure represents OR of a study plotted against the standard error (SE) its OR. Different indicators of the studies belonging to each ancestral group are used in these plots. **Figure S9A**: Begg's Plot for comparisons under dominant genetic model *(4a4a+4a4b vs. 4b4b)*; **Figure S9B**: Begg's Plot for comparisons under recessive genetic model *(4a4a vs. 4a4b+4b4b)*.
(TIF)

Data S1 **The raw data collected from all the published association studies included in the present meta-analysis.**
(ZIP)

Checklist S1 **PRISMA Checklist.**
(DOC)
