# Supplementary figures and images for: Association of Endothelial Nitric Oxide Synthase Gene Polymorphisms with Coronary Artery Disease: An Updated Meta-Analysis and Systematic Review
Source: PLoS One. 2014 Nov 19;9(11):e113363. doi: 10.1371/journal.pone.0113363 (PMC4237457; doi:10.1371/journal.pone.0113363)

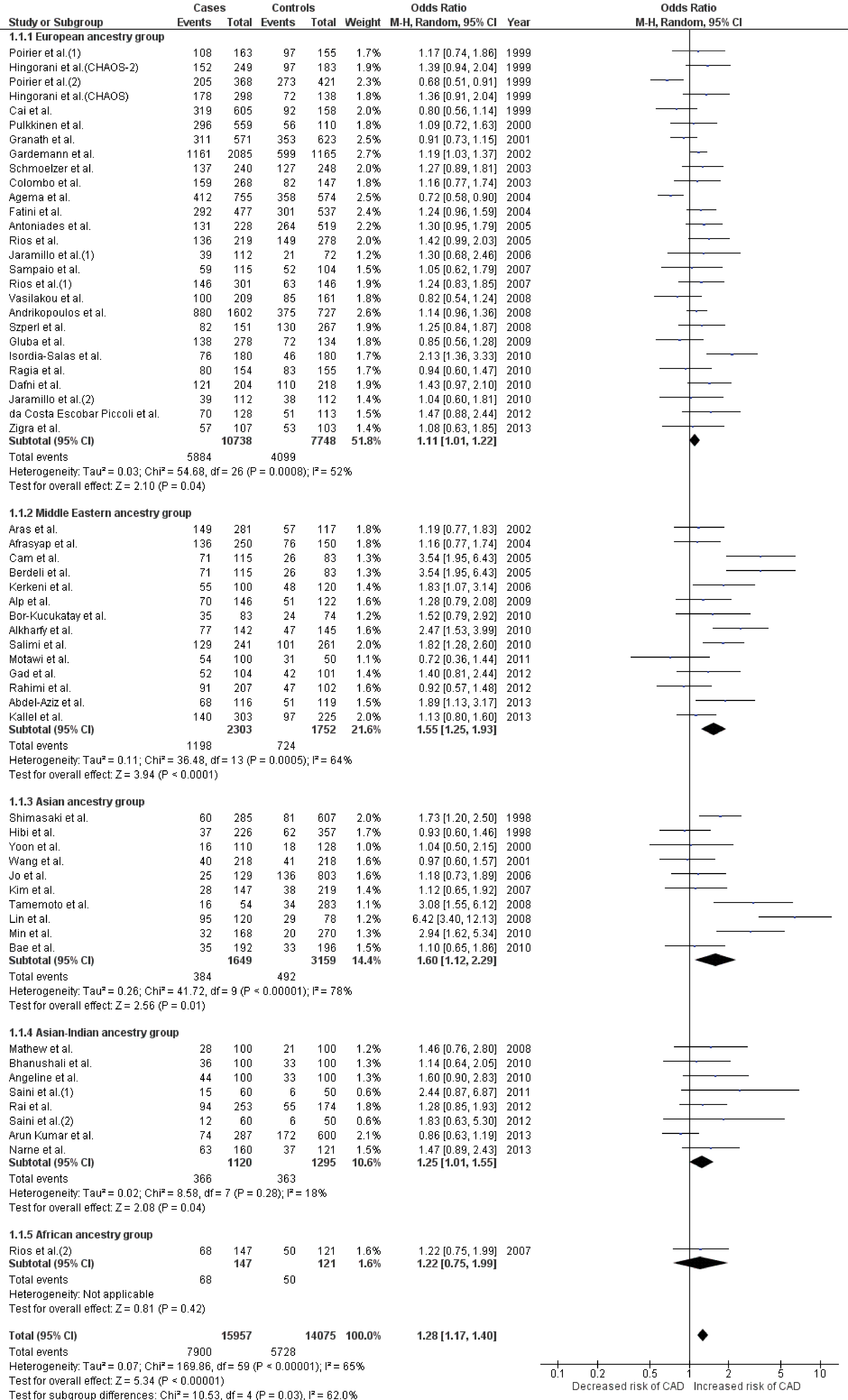

Supplement: Figure S1 — Forest plot depicting results of meta-analysis of studies reporting NOS3 Glu298Asp polymorphism assessed under dominant (TT+GT vs. GG) genetic model. Effect size estimates for all ancestral groups in this plot were obtained using random effects for analysis. Effect sizes using fixed effects were recalculated for Asian-Indian group which showed homogenous distribution among its included studies. Recalculated effect size estimate for Asian-Indians was, OR, 95%CI = 1.21, 1.01–1.45; Z = 2.07; P = 0.04. (TIF) [file pone.0113363.s001.tif]

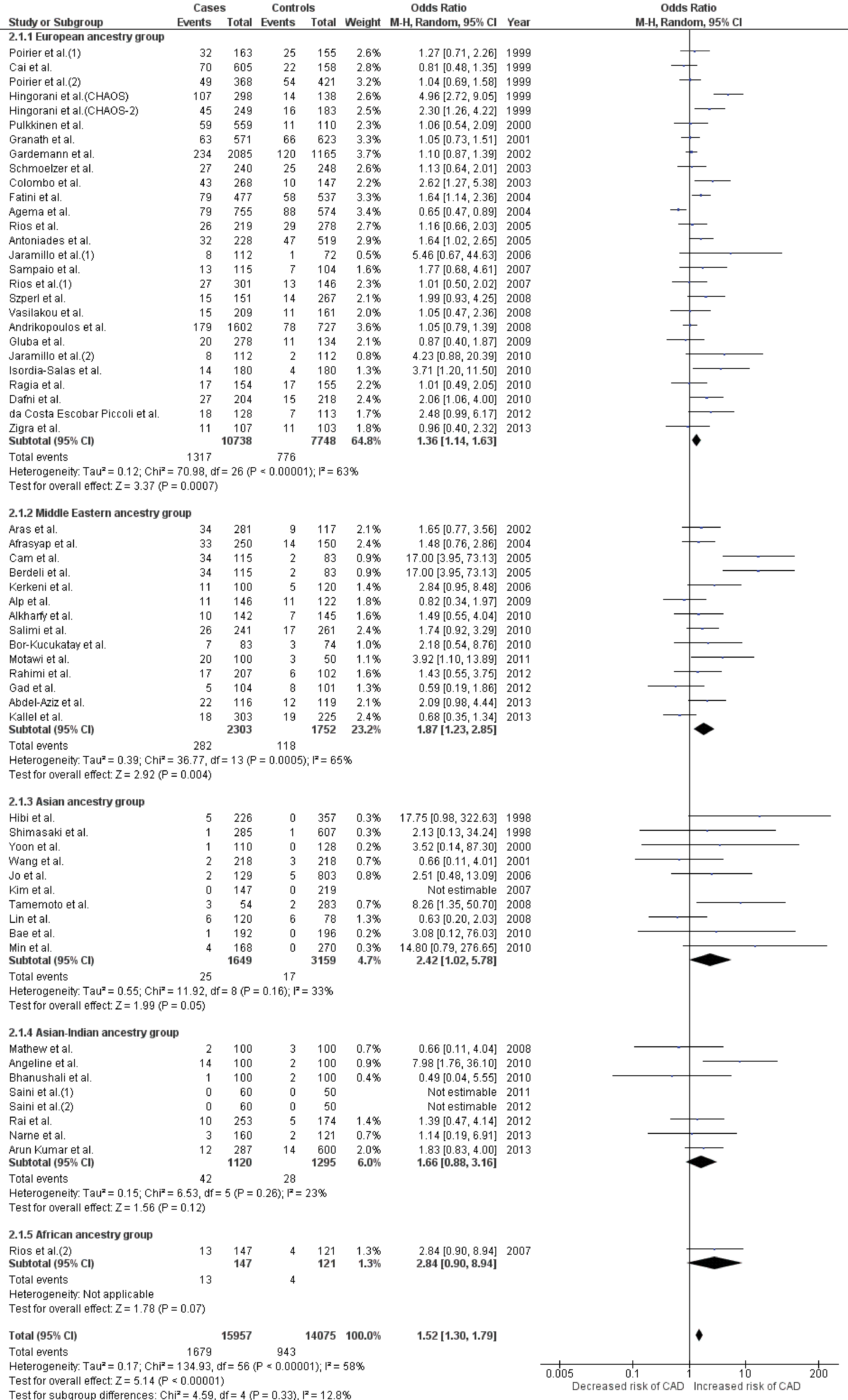

Supplement: Figure S2 — Forest plot depicting results of meta-analysis of studies reporting NOS3 Glu298Asp polymorphism assessed under recessive (TT vs. GG+GT) genetic model. Effect size estimates for all ancestral groups in this plot were obtained using random effects for analysis. Effect sizes using fixed effects were recalculated for Asian and Asian-Indian groups which showed homogenous distribution among its included studies. Recalculated effect size estimates were, OR, 95%CI = 2.22, 1.21–4.08; Z = 2.57; P = 0.01 and OR, 95%CI = 1.85, 1.12–3.04; Z = 2.42; P = 0.02 for Asians and Asian-Indians respectively. (TIF) [file pone.0113363.s002.tif]

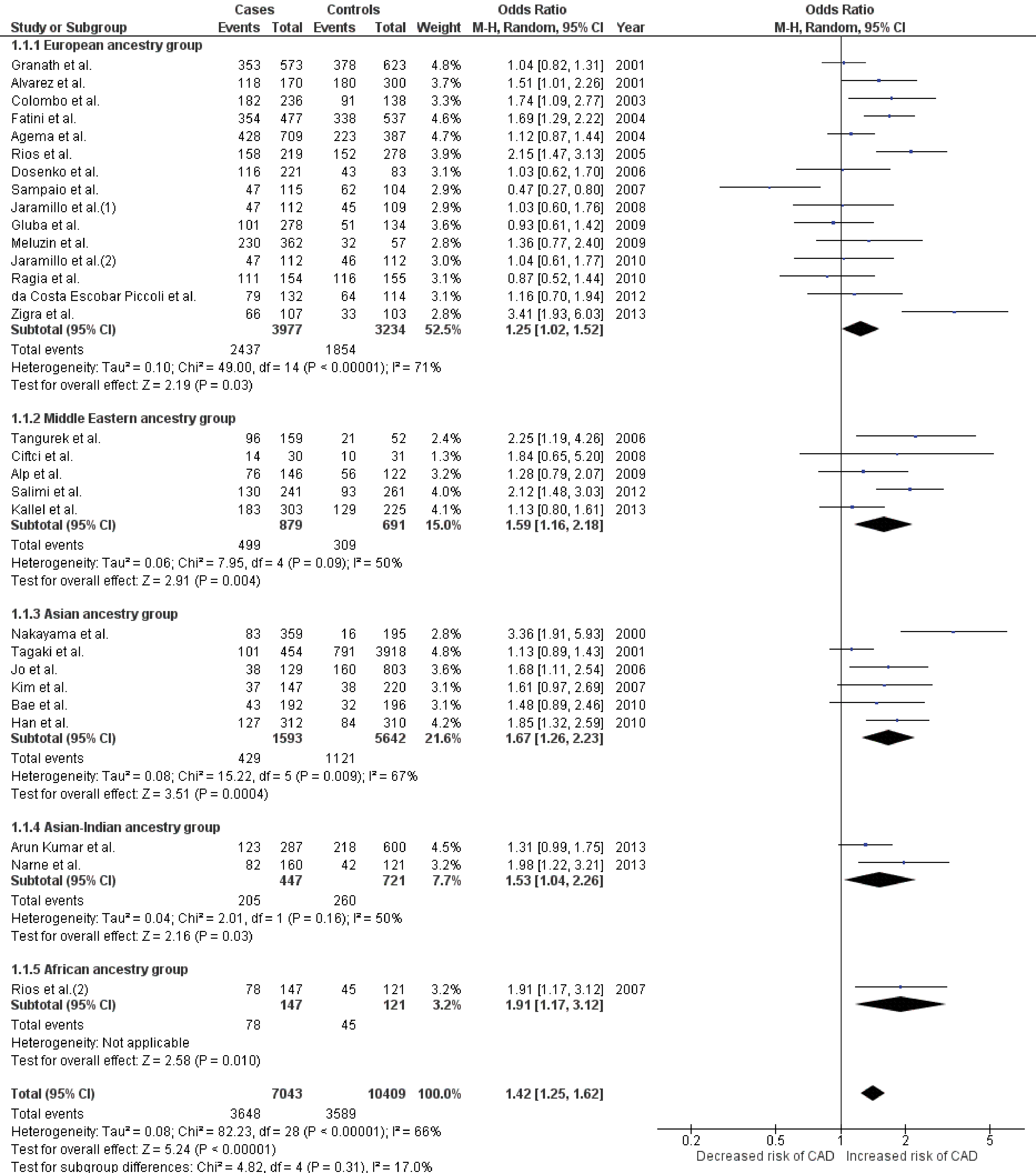

Supplement: Figure S3 — Forest plot depicting results of meta-analysis of studies reporting NOS3 T786-C polymorphism assessed under dominant (CC+CT vs. TT) genetic model. Effect size estimates for all ancestral groups in this plot were obtained using random effects for analysis. (TIF) [file pone.0113363.s003.tif]

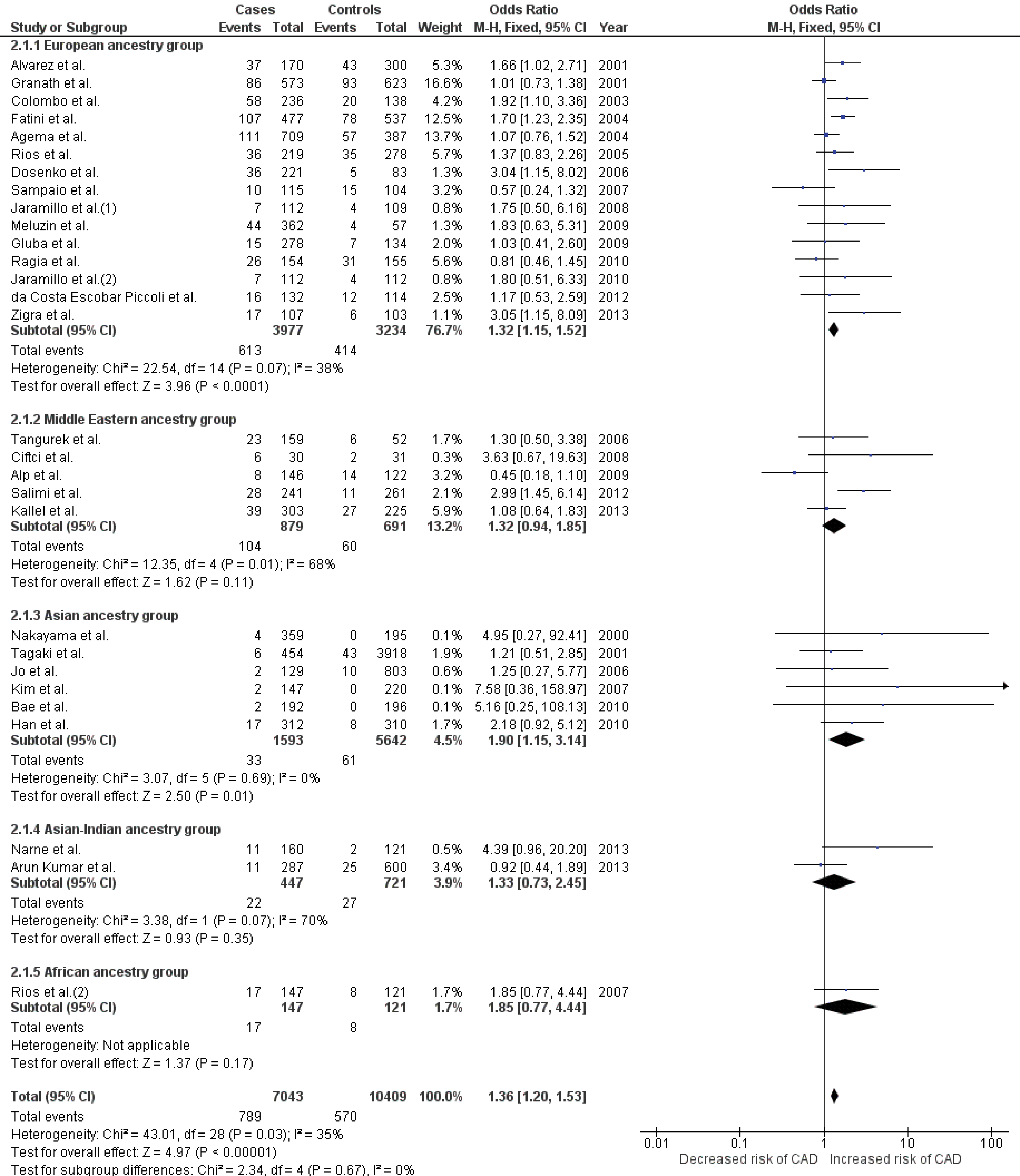

Supplement: Figure S4 — Forest plot depicting results of meta-analysis of studies reporting NOS3 T786-C polymorphism assessed under recessive (CC vs. TT+CT) genetic model. Effect size estimates for all ancestral groups in this plot were obtained using fixed effects for analysis. Effect sizes using random effects were recalculated for three ancestral subgroups viz. Middle Eastern, Asian-Indian and African, which showed homogenous distribution among its included studies. Recalculated effect size estimates were, OR, 95%CI = 1.35, 0.69-2.65; Z = 0.87; P = 0.38 for Middle Easterners; OR, 95%CI = 1.73, 0.38–7.95; Z = 0.71; P = 0.48 for Asian-Indians and OR, 95%CI = 1.85, 0.77–4.44; Z = 1.37; P = 0.17 for Africans. (TIF) [file pone.0113363.s004.tif]

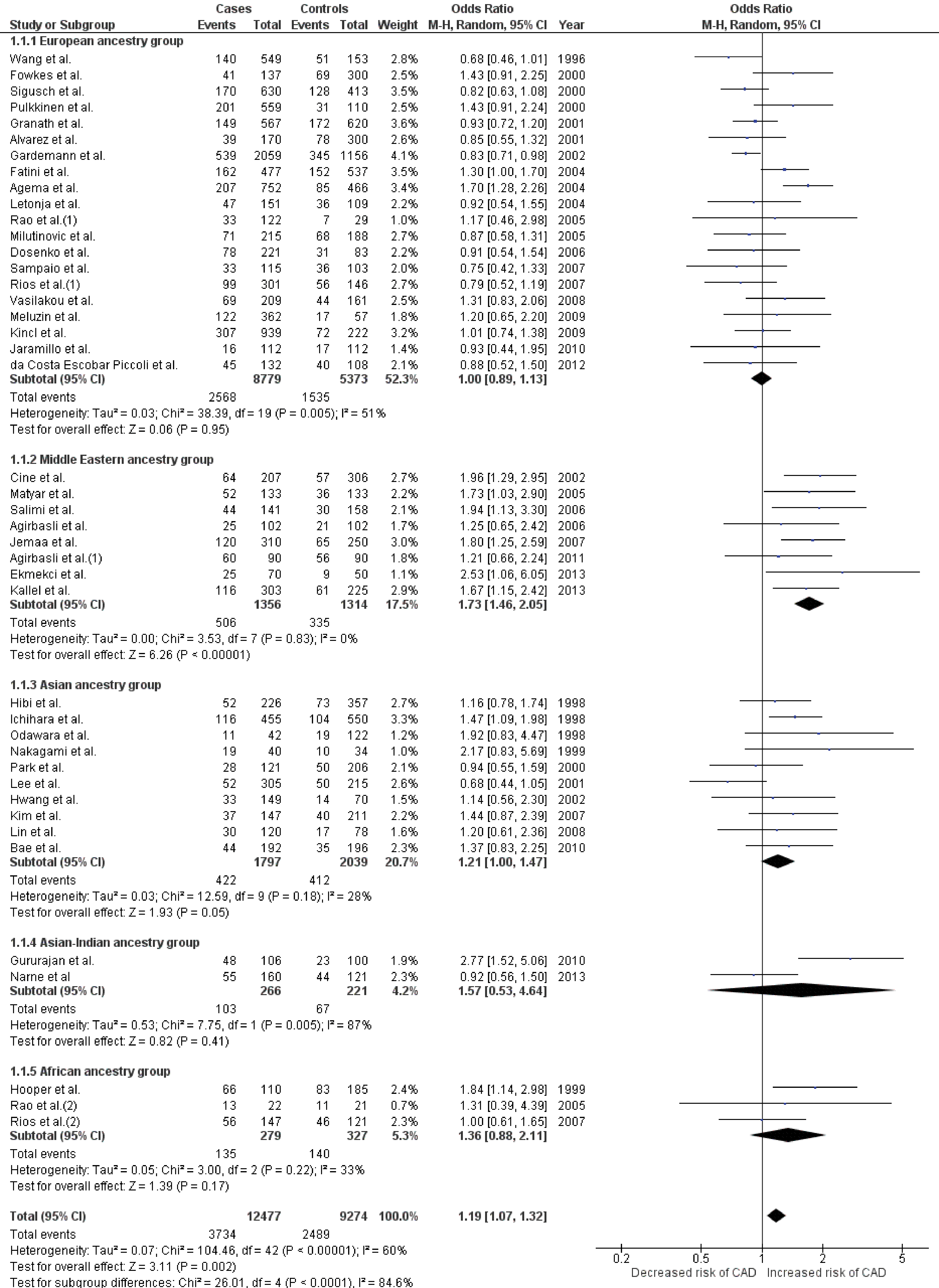

Supplement: Figure S5 — Forest plot depicting results of meta-analysis of studies reporting NOS3 4b/a VNTR polymorphism assessed under dominant (4a4a+4a4b vs. 4b4b) genetic model. Effect size estimates for all ancestral groups in this plot were obtained using random effects for analysis. Effect sizes using fixed effects were recalculated for Middle Eastern and Asian groups which showed homogenous distribution among its included studies. Recalculated effect size estimates were, OR, 95%CI = 1.73, 1.46–2.05; Z = 6.28; P = <0.00001 and OR, 95%CI = 1.22, 1.04–1.42; Z = 2.45; P = 0.01 for Middle Easterners and Asians respectively. (TIF) [file pone.0113363.s005.tif]

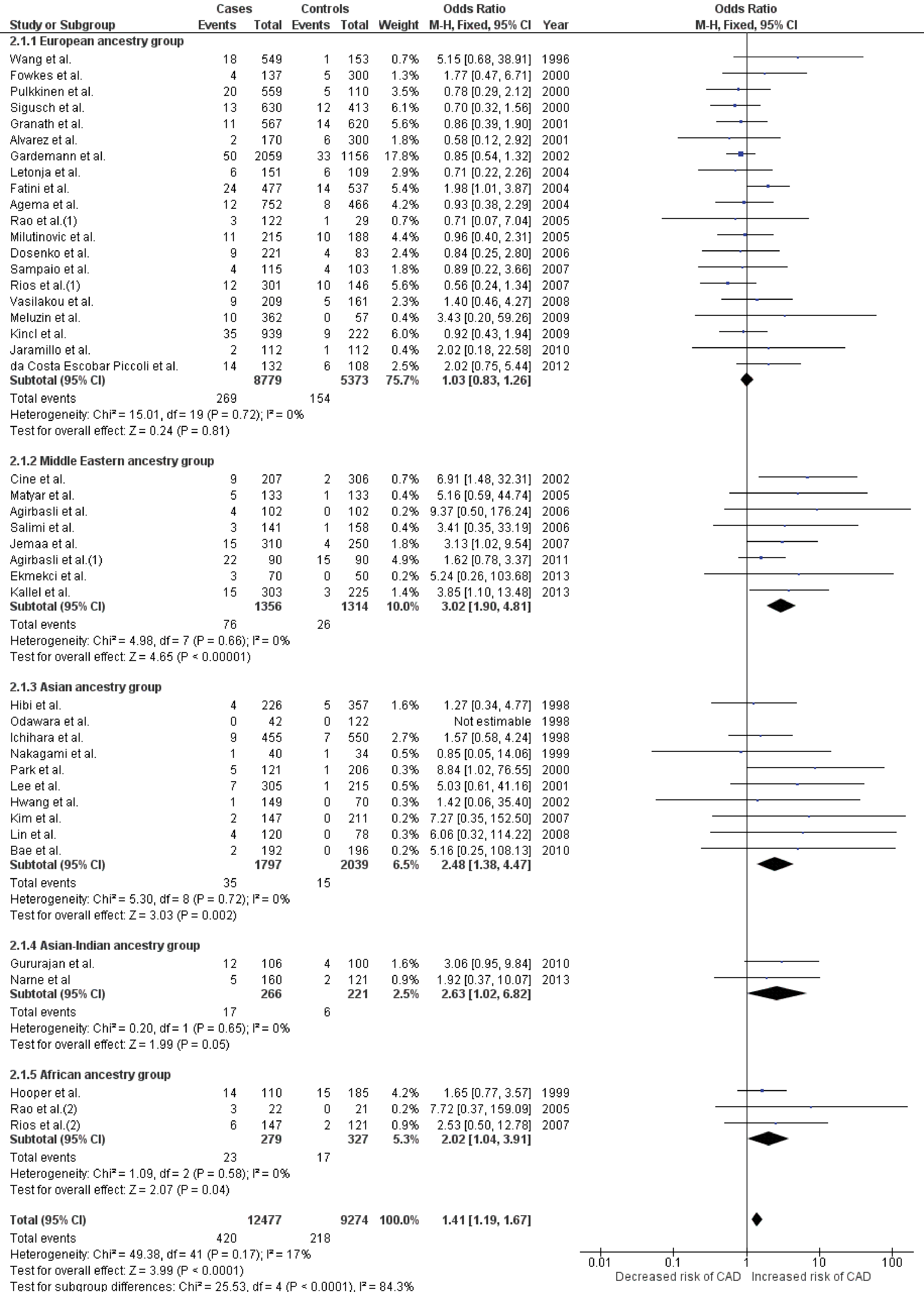

Supplement: Figure S6 — Forest plots depicting results of meta-analysis of studies reporting NOS3 4b/a VNTR polymorphism assessed under recessive (4a4a vs. 4a4b+4b4b) genetic model. Since all the ancestral groups showed homogenous distribution among its included studies, effect size estimates for all groups in this plot were obtained using fixed effects for analysis. (TIF) [file pone.0113363.s006.tif]

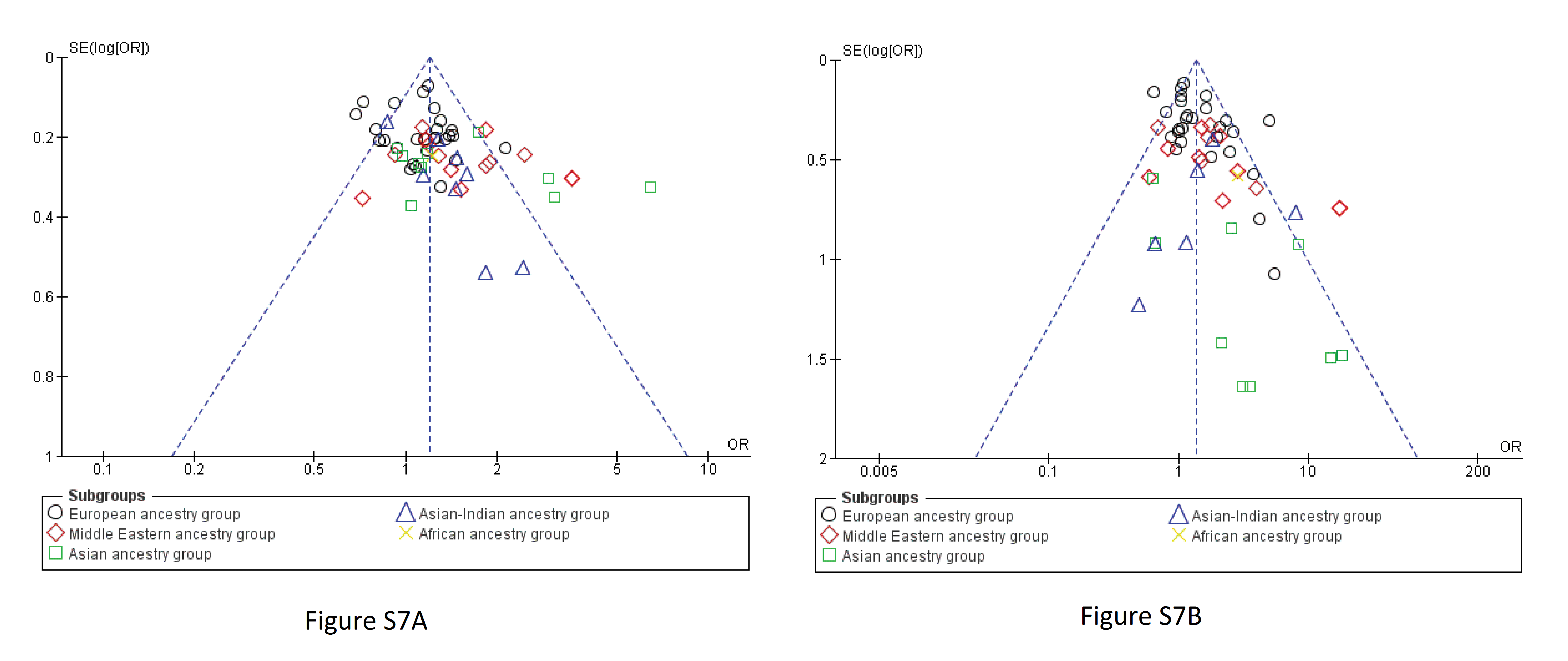

Supplement: Figure S7 — Begg's funnel plots for testing publication bias in comparisons under different genetic models for NOS3 Glu298Asp polymorphism. Each point in each figure represents OR of a study plotted against the standard error (SE) its OR. Different indicators of the studies belonging to each ancestral group are used in these plots. Figure S7A: Begg's Plot for comparisons under dominant genetic model (TT+GT vs. GG); Figure S7B: Begg's Plot for comparisons under recessive genetic model (TT vs. GG+GT). (TIF) [file pone.0113363.s007.tif]

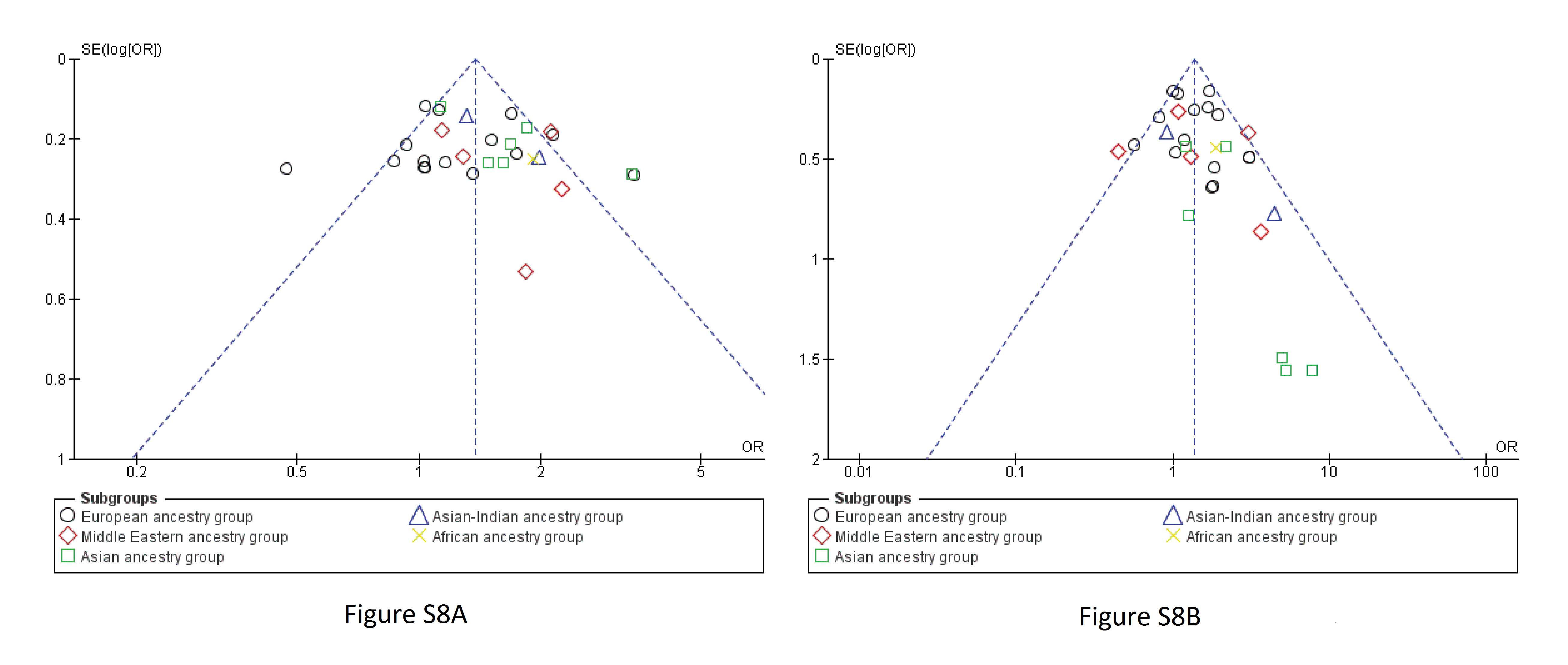

Supplement: Figure S8 — Begg's funnel plots for testing publication bias in comparisons under different genetic models for NOS3 T786-C polymorphism. Each point in each figure represents OR of a study plotted against the standard error (SE) its OR. Different indicators of the studies belonging to each ancestral group are used in these plots. Figure S8A: Begg's Plot for comparisons under dominant genetic model (CC+CT vs. TT); Figure S8B: Begg's Plot for comparisons under recessive genetic model (CC vs. TT+CT). (TIF) [file pone.0113363.s008.tif]

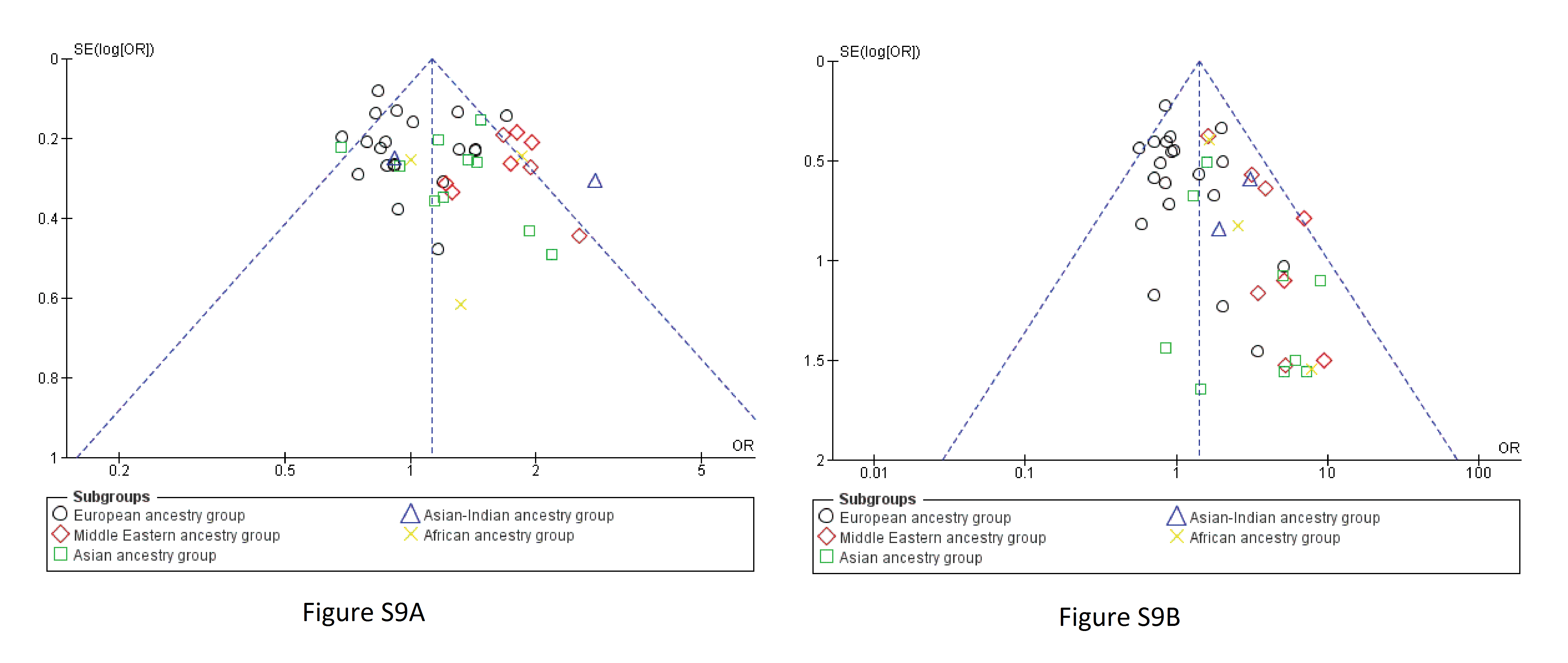

Supplement: Figure S9 — Begg's funnel plots for testing publication bias in comparisons under different genetic models for NOS3 4b/a VNTR gene polymorphism. Each point in each figure represents OR of a study plotted against the standard error (SE) its OR. Different indicators of the studies belonging to each ancestral group are used in these plots. Figure S9A: Begg's Plot for comparisons under dominant genetic model (4a4a+4a4b vs. 4b4b); Figure S9B: Begg's Plot for comparisons under recessive genetic model (4a4a vs. 4a4b+4b4b). (TIF) [file pone.0113363.s009.tif]
